# State of the evidence 2017: an update on the connection between breast cancer and the environment

**DOI:** 10.1186/s12940-017-0287-4

**Published:** 2017-09-02

**Authors:** Janet M. Gray, Sharima Rasanayagam, Connie Engel, Jeanne Rizzo

**Affiliations:** 1 Department of Psychology and Program in Science, Technology, and Society, Vassar College, 124 Raymond Avenue, Poughkeepsie, NY 12604-0246 USA; 2 Breast Cancer Prevention Partners, 1388 Sutter St., Suite 400, San Francisco, CA 94109-5400 USA

**Keywords:** Breast cancer, Environmental toxicants, Endocrine disrupting compounds, Bisphenol a, Light-at-night, Radiation

## Abstract

**Background:**

In this review, we examine the continually expanding and increasingly compelling data linking radiation and various chemicals in our environment to the current high incidence of breast cancer.

**Abstract:**

Singly and in combination, these toxicants may have contributed significantly to the increasing rates of breast cancer observed over the past several decades. Exposures early in development from gestation through adolescence and early adulthood are particularly of concern as they re-shape the program of genetic, epigenetic and physiological processes in the developing mammary system, leading to an increased risk for developing breast cancer. In the 8 years since we last published a comprehensive review of the relevant literature, hundreds of new papers have appeared supporting this link, and in this update, the evidence on this topic is more extensive and of better quality than that previously available.

**Conclusion:**

Increasing evidence from epidemiological studies, as well as a better understanding of mechanisms linking toxicants with development of breast cancer, all reinforce the conclusion that exposures to these substances – many of which are found in common, everyday products and byproducts – may lead to increased risk of developing breast cancer. Moving forward, attention to methodological limitations, especially in relevant epidemiological and animal models, will need to be addressed to allow clearer and more direct connections to be evaluated.

## Background

In this review, we examine the continually expanding and increasingly compelling data linking radiation and various chemicals in our environment to the current high incidence of breast cancer. We acknowledge the importance of many widely understood risk factors for breast cancer including: primary genetic mutations, reproductive history, and lifestyle factors such as weight gain, alcohol consumption and lack of physical exercise [1, 2]. Yet we begin with an understanding that in total, these factors do not address a considerable portion of the risk for the disease [2–5]. A substantial body of scientific evidence indicates that exposures to common chemicals and radiation, singly and in combination, also contribute to the increasingly high incidence of breast cancer observed over the past several decades. Although rates have leveled off overall in the past few years for some subsets of women, there was a significant and progressive rise in the incidence of breast cancer in the decades following World War II [6, 7], the same decades that saw exponential increases in the use of chemicals for production of pesticides, herbicides, plastics, cosmetics and other commonly used materials and products [8–10].

This report focuses on these environmental issues. In the 8 years since we last published a comprehensive review of the relevant literature [11], hundreds of new papers have been published supporting this link, and the evidence on this topic is more extensive and of better quality than that previously available. After describing our methodology for selecting scientific reports and reporting of statistical findings, we present introductory sections on breast cancer statistics and subtypes as well as critical concepts for framing the complex data we are exploring. We then examine the literature on exposures to environmental toxicants and risk for developing breast cancer, dividing the evidence discussion into seven major sections: (1) Hormones: Pharmaceutical agents & personal care products; (2) Endocrine disrupting compounds (EDCs); (3) Hormones in food: Natural and additives; (4) Non-EDC industrial chemicals; (5) Tobacco smoking: Active and passive; (6) Shift work, light-at-night and melatonin; and (7) Radiation. We conclude with a brief synopsis and reflection on the state of the evidence, including methodological limitations and promises, as well as directives for future research needs.

## Methodology

### Article selection process

The goal of this review is to offer a broad overview of the scientific literature examining the potential connections between exposure to environmental toxicants and changes in the risk for developing breast cancer, updating our last review of this topic published in 2009. To fully incorporate the relevant materials, we entered the following search terms into both PubMed and Scopus: ‘breast cancer’ and ‘mammary tumors’ in conjunction with ‘environment’, ‘endocrine disruptors/endocrine disrupting compounds’ and all of the individual toxicants covered in this report.

In selecting epidemiological studies, we emphasized work from the past 10 years. When studies were follow-up reports from large, longitudinal studies, we also reported the earlier data as the length of time between exposures and outcome assessments could lead to different conclusions, or recognition of different results as the participants in the study reached later ages and, especially, progressed from pre-menopausal to post-menopausal status.

Over the 8 years since our last report, there has been a substantial increase in the amount of information focused on mechanisms underlying the complex relationships between exposures and risk for developing breast cancer. This is especially true in the growing field examining exposures to endocrine disrupting compounds and disease risk. We therefore focused on articles from the past 8 years. While we did not report every gene whose expression might be affected by a particular exposure, we did try to give a full overview of the current understanding of physiological, developmental, genetic, epigenetic and endocrine processes that are affected by exposures relevant to a change in risk for developing breast cancer. Although the emphasis was on the most recent data, we included earlier results when they were needed as background or to provide a fuller picture of the evidence.

Exceptions to our primary reliance on very recent literature are found in the sections on non-endocrine disrupting industrial chemicals and some pesticides and herbicides. Much of the relevant data for these toxicants comes from studies from 25 to 30 years ago, when the National Toxicology Program (NTP) and International Agency for Research on Cancer (IARC) were determining possible carcinogenicity of these chemicals.

Finally, in selecting studies to report, we took care to include studies that had negative results, that is, those that reported no significant relationship between exposures and risk for developing breast cancer. Where possible, we then explored possible differences in study design or methods that might account for differences in results across studies.

### Reporting of statistics for epidemiological studies

We report the statistics (e.g., RR, OR, HR, etc.), along with 95% confidence levels, as offered by the authors of the individual reports. Where explicit adjustments were made, we note the type of statistic used and the variable of adjustment. More often though, factors including age, menopausal status, breast cancer subtype (by receptor status, ductal vs. lobular, in situ vs. invasive, etc.), racial/ethnic identity, are reported as main factors to be analyzed, along with effects of particular exposures. Significant main effects and interactions between exposures and these other variables are reported in this review.

## Introduction

In this introductory section, we provide basic statistics and a brief exploration of the several subtypes of breast cancer – recognizing that the term ‘breast cancer’ is often used as a proxy for several distinct genetic, histopathological, and hormonal profiles for the disease. We then introduce a series of key framing concepts necessary for appreciating the complex evidence supporting (or not) a growing understanding of the data implicating specific environmental toxicants in an increased risk for developing breast cancer. These framing concepts include: (a) low-dose and non-monotonic responses; (b) interactions between environmental toxicants; (c) gene-environment interactions and epigenetic changes; (d) cell-cell interactions and the Tissue Organization Field Theory; and (e) timing of exposures. We conclude with a schematic model of the complexity of factors influencing risk for developing breast cancer, with an emphasis on environmental factors.

### Breast cancer statistics

The Surveillance, Epidemiology, and End Results (SEER) program of the National Cancer Institute (NCI) predicted that in 2015 in the U.S., 40,290 women and 440 men would die of breast cancer and 231,840 women and 2350 men would be diagnosed with invasive breast cancer; another 60,290 women would be diagnosed with breast cancer in situ. As of early 2016, the NCI estimated that approximately 3,560,570 U.S. women are living with a prior diagnosis of breast cancer [12].

The most recent year for which accurate data exist related to breast cancer incidence and mortality is 2012. In addition to total national incidence and mortality reports, SEER data are broken down by major census self-described categories of race/ethnicity. The average incidence rates (number of women diagnosed per 100,000 women, age-adjusted and normalized to the 2000 standardized U.S. population) across the 5 years from 2008 to 2012 differed across census categories, as did the trends across time. Five-year average incidence rates for whites were the highest (126.1), with rates for black women only slightly lower (124.1). However, in 2012 for the first time since SEER began collecting data in 1975, incidence for these two groups converged; historically black women had a significantly lower rate of the disease. Average 5-year incidence rates were lower for American Indian/Native American (91.9), Hispanic (91.9) and Asian-Pacific Island (88.3) women [12].

Across racial and ethnic groups in the U.S., mortality rates (deaths per 100,000 women, age-adjusted and normalized to the 2000 standardized U.S. population) from breast cancer have decreased since their peak in the mid-late 1990s. Despite this apparent good news, significant racial/ethnic disparities have remained consistent over the last several decades. In the U.S., black women have the highest breast cancer mortality rate (31.0) of any racial/ethnic group. Asian/Pacific Islander women have the lowest mortality rates (11.4), with white (21.9), Hispanic (14.5) and American Indian/Native American (15.0) women having intermediate rates. Despite the universal drop in mortality rates across the past two decades and the similarity in incidence rates, over the same time period the disparities between mortality rates for white and black women have grown significantly; the mortality rate for black women diagnosed with breast cancer is 42% higher than the comparable rate for white women [12, 13].

### Breast cancer subtypes

Breast cancer is not a singular disease, and it will be important throughout this report to examine, where possible, the subtype(s) of the disease most affected by exposures to environmental toxicants. Several classification systems have been developed to distinguish different subtypes of the disease including age of patient (usually split by pre or post-menopausal, with age 50 often as the proxy for the shift between reproductive phases); in situ, localized, regional or metastatic presentation; morphological characteristics; histological grade and cellular proliferation rate; or gene expression profile [14–17].

Of particular relevance to the discussion of environmental exposures, especially to endocrine disrupting compounds (EDCs), is the classification based on expression of the estrogen receptor (ER), progesterone receptor (PR) or the HER2 oncogene. Two luminal subtypes (A and B) express ER but not HER2, with Luminal A co-expressing PR and having a low proliferation rate and Luminal B having either high proliferation rate or low PR expression. Luminal B-like (HER2 positive) expresses ER and high HER2 levels, with any proliferation and PR profile. The HER2 positive subtype has overexpression of HER2 but without ER or PR being present. Triple negative breast cancer has no expression of ER, PR or HER2 [18].

Breast cancer subtypes are not randomly distributed across the population and there are differences found when diagnoses are stratified by age, race/ethnicity, reproductive history, body mass index, socioeconomic status, or geographical location [17, 19–22]. For example, younger women in general, and younger black women in particular, are more likely to present with the triple negative (ER-, PR-, and HER2-) subtype of the disease, a diagnosis that is both more aggressive and less responsive to treatment than ER+/PR+ or HER2+ tumors [12, 23, 24]. Like young black women, Latinas are also disproportionately affected by aggressive triple-negative tumors [17, 24, 25].

### Race and ethnicity

The existence of differences across self-identified race and ethnic categories do not necessarily imply genetic differences. Indeed, they reflect the complexity of geographic location; social and socioeconomic status; personal and community stress and security; lifestyle factors including diet, exercise, alcohol and pharmaceuticals use; physiological responses to life factors; gene-environment interactions; and epigenetic changes — all factors that may change over the lifetime of the individual and may vary considerably among people who self-identify in a particular race/ethnicity category [26, 27]. Because of the confounds of economic and social factors, people of different racial/ethnic identities may also experience different environmental and occupational exposures to disease-affecting toxicants [28–30].

Racial and ethnic minorities often are exposed to disproportionately high levels and varieties of environmental pollutants in the U.S. [31], as are people living in poverty [32]. There are racial/ethnic differences in the body burden of different environmental chemicals that have been associated with increased risk for breast cancer. Blacks have higher body burden levels than whites or Mexican Americans of many chemicals including many polychlorinated biphenyls (PCBs), mercury, polyaromatic hydrocarbons (PAHs), and phthalates. Mexican Americans have higher levels of the pesticide dichlorodiphenyltrichloroethane (DDT) [33]. Varying body burdens of some chemicals including bisphenol A (BPA), polyfluorinated chemicals (PFCs) and triclosan, all commonly found in household products, are associated with both race/ethnicity and socioeconomic status [27, 34, 35]. Yet as Nelson points out, socioeconomic status and race/ethnicity most probably serve independently as markers for other activities or circumstances that influence the level of exposures to potentially toxic chemicals [27].

### Framing concepts

Building on and extending the ‘Hallmarks of Cancer’ framework proposed by Hanahan and Weinberg [36], an international team of 170 scientists participating in the Halifax project recently evaluated the contributions to carcinogenesis of low-dose exposures to individual compounds and mixtures of environmental chemicals on each of the proposed hallmark phenotypes [37]. Other recent reviews have focused on the importance of evaluating: non-monotonic dose-response relationships, especially between EDCs and health outcomes [38]; timing of exposures to environmental toxicants, with an emphasis on fetal to adolescent exposures to EDCs and later development of diseases [39–41]; environmental carcinogenesis from the perspective of disruptions of cell-cell (e.g., stromal-epithelia) interactions [42, 43]; gene-environment interactions [44, 45]; the importance of using the principles of basic endocrinology in establishing mechanistic models for examining health impacts of exposures to EDCs [46, 47] and the relevance for these mechanisms in understanding the growing appreciation of the links between environmental toxicants and increased risk for many diseases, including breast cancer [48–52].

In this current paper, we will not offer comprehensive overviews of these framing concepts, but refer the reader to the reviews cited above. Instead we will briefly introduce the main concepts with a couple of examples relevant to exploring the following evidence linking exposures to environmental chemicals toxicants with increased risk for development of breast cancer. While some of the chemicals of concern are traditionally defined carcinogens, many more fall into the class of endocrine disrupting compounds (EDCs), a group of exogenous compounds that exert at least part of their impacts on health outcomes by altering the activity of the endocrine system.

#### Low-dose and non-monotonic responses

EDCs disrupt the endocrine system. As such, their mechanisms of action and properties are different than most non-EDC carcinogens for which the toxicological model is that higher doses are more damaging than are lower doses; the relationship between dose and damage is functionally linear; and there may be safe levels below which no negative impact is observed (the No-Observed-Adverse-Effect Level or NOAEL) [53, 54]. Instead, in many ways, EDCs act much as natural hormones do: at very low doses, especially during critical periods of development, and often following non-monotonic response (NMRs) curves [38, 47]. Thus sub-cellular and physiological responses to low dose exposures may be greater than, or at least different from, exposures to higher doses.

For example, many animal studies have demonstrated that prenatal or neonatal exposures to bisphenol A (BPA) lead to changes in mammary tissue development that increase the likelihood of the later development of mammary tumors. Yet some of these effects are dependent on dose, but not in a linear fashion. In one study, prenatal exposures to low (and environmentally relevant) doses of BPA had significant effects on the mammary gland gene expression profile just prior to the onset of puberty, while higher exposure levels altered expression of different genes and at a much later age [55]. In another report, rat dams were exposed via gavage to no BPA, or doses ranging from 0.025 to 50 mg BPA/kg bw/d from day 7 of gestation through weaning of their pups. As adults, female offspring that had been exposed to the 0.25 mg dose had increased incidence of intraductal hyperplasia, although no similar effects were found for either higher or lower exposures [56].

Blei et al. examined the lifelong effect of dietary exposures to two different amounts of soy-derived isoflavones, choosing doses that yielded concentrations similar to the highest and lowest plasma levels of isoflavones in Asian women. Although both low and high exposure levels led to an earlier onset of puberty, only low levels of exposure led to increased expression of the proliferation marker Ki67 in mammary glands of 97-day old adults. On the other hand, only higher exposure levels led to significant decreases in the expression of the proliferation marker PCNA in mammary tissue from ovariectomized rats that had been treated with estradiol. In these animals, estradiol administration led to additive stimulation of PR induction in animals that were exposed to the low dose exposures, while the high dose exposure levels inhibited the estradiol-induced expression of PR in mammary gland [57].

#### Interactions between environmental toxicants

Numerous animal studies indicate that the kinds of mixtures to which an animal is exposed matter in determining ultimate risk [58]. Only a relatively few combinations and doses of chemicals have been tested. This is perhaps not surprising: One estimate predicts that it would require 166 million experiments to test all combinations of three out of the 1000 most common synthetic chemicals currently in use [59]. While only a small portion of those studies have actually been conducted, there are several reports demonstrating that mixtures of environmental chemicals or chemicals and radiation, may alter biological processes and possibly lead to increases in breast cancer risk.

For example, the E-screen assay uses ER+ human breast cancer tumor cells (MCF-7 cells) that are dependent on estrogens for cell growth and proliferation [60], and single studies can examine the effects of scores of chemicals at multiple doses, alone and in combination on breast cancer cell proliferation [61, 62]. An examination of the combined effects of 11 different environmental contaminants – all added at NOAEL concentrations – showed that the chemicals had additive effects with each other and also with naturally occurring estradiol [63]. At levels found in our environment, the ubiquitous plasticizer bisphenol A also significantly increased the effects of estradiol [64].

Payne et al. used the yeast estrogen screen (YES), an in vitro assay of estrogen receptor activation, to examine the combined effects of a pesticide residue (o,p’-DDT), a plant estrogen (genestien, found in soy) and two alkylphenol surfactants (sudsing agents and chemical dispersers; 4-n-octylphenol and 4-nonlyphenol). Clear additive effects of the four chemicals were found [65].

Rivero et al. examined the effects of two mixtures of organochlorine pesticides, the first composed to mimic the chemical profile found in healthy women and the second to mimic the pesticide profile found in breast cancer patients. Both mixtures down-regulated genes whose expression is involved in the binding of ATP in normal human mammary epithelial cells, but there were very different effects of the two mixture profiles on the expression of oncogenes and tumor suppressor genes [66, 67]. Similarly, combinations of different organochlorine pesticides, mixed to mimic combinations found in human samples, increased cytotoxic effects in a cell line derived from normal human breast epithelial cells [68].

In a study of mammary tissue development, mixtures of chemicals commonly found in the environment made rat mammary tissue more susceptible to exposures to dietary estrogens after birth, leading to tissue abnormalities that have been associated with mammary tumors [69]. And pre-treatment of young rats with a low dose of radiation resulted in earlier occurrence and increased frequency of mutated mammary tumors after subsequent exposure to a known chemical carcinogen [70].

#### Gene-environment interactions and epigenetic changes

Several studies have reported an increased risk for developing breast cancer in women with either *BRCA1* or *BRCA2* mutations following exposure to medical radiation, either through mammography or radiation therapy [71–74]. Another report found that a combination of multiple variants in genes associated with DNA repair mechanisms led to an increase in mammography-associated risk for developing breast cancer [75].

Other studies have reported an interaction between various gene variants associated with breast cancer risk and exposures to environmental exposures [76]. But overall, the relevant literature is mixed, with different single-nucleotide polymorphisms (SNPs) and different environmental toxicants being tested. A comprehensive overview of the field concluded that these studies were too few and underpowered for any clear demonstration of interactions between particular SNPs or clusters of SNPs and environmental factors in affecting breast cancer risk, given that most large epidemiological studies yield, at best, very small effects that are often nonreplicable [45]. Nevertheless, the authors concluded that, ‘Presently, we should consider hereditary variants and environmental factors as multiplicative/additive factors in the prediction of breast cancer risk’ [45].

In addition to genetic polymorphisms influencing the effects of environmental toxicants on inter- and intracellular responses, environmental chemicals, especially EDCs, can alter the regulation of genes involved in cell proliferation, apoptosis signaling pathways, etc. through epigenetic processes [77, 78]. Through mechanisms including altered DNA methylation, modifications of histones and expression of small regulatory RNAs (microRNAs), chemical and radiation exposures can have profound effects on the structure and function of the developing mammary gland [79–82].

For example, Kutanzi and Kovalchuk reported that concurrent treatment of adult ACI rats with exogenous sources of estradiol and radiation resulted in increased mammary gland methylation and acetylation of H3 and H4 histones, and significantly increased induction of MAPK and p38 pathways, known biomarkers for chromosome instability [83]. And in normal MCF-7 human ER+ cell line, addition of the growth promoter, zeranol, led to stimulatory effects on cell growth. These results were driven, at least in part, by down-regulation of the tumor suppressor gene p53, a process that was accompanied by up-regulation of DNA-methyltransferase 1 [84].

Hussain et al. explored the effects of BPA on the expression of HOXC6, a homeobox-containing gene that is associated with mammary cell growth and development and which is overexpressed in many breast cancers. Both in MCF-7 cell lines and in mammary tissue from adult Sprague-Dawley ovariectomized rats, BPA exposure increased histone methylation and acetylation and recruited RNA polymerase II at the HOXC6 promoter, resulting in HOXC6 overexpression [85]. Similarly, Doherty et al. demonstrated in both MCF-7 cells and in mammary glands from neonatally exposed mice that either BPA or diethylstilbestrol (DES) treatment led to a 2–3 fold increase in expression of the breast cancer associated histone methyltransferase, Enhancer of Zeste Homolog 2 (*EZH2*) mRNA expression and subsequent EZH2 synthesis. These changes were accompanied by increased trimethylation of histone H3, both in vivo and in vitro [86].

#### Cell-cell interactions and the tissue organization field theory

Rather than modeling cancer development as a result of accumulated DNA mutations, with consequent hallmark changes in cell physiology building on the initial genetic instability [36, 37], the Tissue Organization Field Theory (TOFT) of carcinogenesis [87, 88] is based on a more ecological view of cellular functioning and tissue organization. TOFT begins by recognizing that cell proliferation is the default state for cells, with processes and chemical signals critically regulating the rate of proliferation, and also that cells work in constant interaction with neighboring cells in the various tissues within an organ [87]. Perturbations of the reciprocal signals and disruption of cell-to-cell interactions, specifically between the mesenchyme/stroma and the parenchyma/epithelial compartments of the developing mammary gland, may underlie the development of breast cancer [39].

Much of the work exploring this model has been done examining the effects of prenatal or neonatal exposure to BPA and morphological changes in the stromal and epithelial compartments of the rodent mammary gland [40, 89–92]. For example, Wadia et al. explored the effects of low dose prenatal exposures to BPA on morphological changes in fetal mouse mammary glands using exposure levels that have previously been demonstrated to induce pre-neoplastic and cancerous tumors in adulthood. Neonatal BPA exposures led to changes in gene expression in both the epithelial and stromal compartments of developing mammary glands from gestational day 19 mice. Altered expression in the stromal fraction was found for genes involved in pathways mediating focal adhesion and adipogenesis, while in the epithelial fraction there were changes in expression of genes involved in apoptosis. Resulting morphological changes due to BPA exposure included advanced fat pad development and delayed epithelial lumen formation, effects that are eliminated in the absence of ERα. Together these data led the authors to propose that BPA (and estrogens, more generally) act directly on the stroma where prenatal estrogen receptors (ERα, ERβ, and GPR30) are expressed. In turn, signals from the stroma alter epithelial gene expression and, ultimately, the earliest morphological programming for the developing mammary gland [89].

#### Timing of exposures

A large body of research demonstrates that the timing of exposures across the lifespan can have an enormous influence on whether, how much, and how an environmental exposure might influence the risk for later development of breast cancer. Mammary cells are more susceptible to the carcinogenic effects of hormones, chemicals and radiation during early stages of development, from the prenatal period through puberty and adolescence, and on until the first full-term pregnancy. Particular concerns have been demonstrated for exposure during prenatal and early childhood periods. Much of this data comes from the use of animal models (reviewed in appropriate sections within this report), but there also are several sources of data that support this claim from the human clinical literature.

For example, daughters of mothers who suffered from preeclampsia during pregnancy, associated with lower levels of maternal estrogens, have decreased risk of developing breast cancer in adulthood [93, 94]. At birth, umbilical cord levels of estriol (E3) and estetrol (E4) – but not estradiol (E2) or estrone (E1) – have been shown to be lowered in neonates delivered from pregnancies associated with preeclampsia [95]. On the other hand, girls who are born with lower birth weight, associated with higher fetal estrogen exposures, have increased risk of later breast cancer diagnosis [96, 97].

And although it is rare to have exposure to exogenous chemicals only during fetal development, between 1938 and 1971 millions of fetuses were exposed to the synthetic estrogen, diethylstilbestrol (DES), when their pregnant mothers were prescribed the drug in order to prevent miscarriages and other complications of pregnancy. DES was banned when daughters of women who took the drug during pregnancy were found to have increased rates of an extremely rare clear-cell vaginal adenocarcinoma. DES exposure was also associated with an increased risk of breast cancer in the mothers [98–100].

In a follow-up study of daughters who were exposed prenatally to DES, a nearly twofold increase in breast cancer risk was observed in women older than age 40. An even greater effect was found for women over the age of 50, although relatively few of the daughters had yet reached that age at the time of the study [101, 102]. Women exposed in utero who had the most severe abnormalities of their vaginal epithelial cells (an indicator of exposures to higher doses of DES) also had a higher risk for developing breast cancer [99]. It now appears that granddaughters of women prescribed DES during pregnancy are also experiencing an elevated incidence of breast cancer [100].

In a case-control prospective study of 9300 women in a pregnancy cohort, stored postpartum maternal blood samples were analyzed for levels of dichlorodiphenyl-trichloroethane (DDT). Daughters were followed for 52 years and breast cancer diagnosis in this cohort was determined. Higher maternal DDT levels were associated with an almost 4-fold increase in occurrence of breast cancer in their daughters by age 52 [103].

A prospective, nested case-control study of 258 women explored their estimated historical DDT levels based on aggregate data from their year of birth as well as blood DDT levels at the time the women gave birth to their first child. Exposure to DDT during childhood and early adolescence (younger than 14 years) was associated with a 5-fold increase in the risk of developing breast cancer before age 50. The younger the women were when the heavy use of DDT was begun in 1945, the greater the risk [104].

Other studies have demonstrated that childhood and adolescence are particularly susceptible ages for exposure to medical radiation and later development of breast cancer. Decades of research have confirmed the link between radiation and breast cancer in women who were irradiated for many different medical conditions, including tuberculosis [105], benign breast disease [106, 107], acute postpartum mastitis [108], enlarged thymus [109, 110], skin hemangiomas [111], scoliosis [112], Hodgkin’s disease [113–116], non-Hodgkin’s lymphoma [117], acne [118], and prophylactic dental care [119]. Evidence from almost all conditions suggests that exposure to ionizing radiation during childhood and adolescence is particularly dangerous with respect to increased risk for breast cancer later in life [73, 120, 121].

*Section summary:* These framing concepts reveal the complexity of research examining relationships between environmental toxicants and risks for developing breast cancer. Breast cancer does not present with a single biomarker profile; incidence rates differ across ethnic/racial and resource-level groups; concentrations of exposures may make a difference, as do possible mixtures and interactions. And specific timing and duration of exposures, especially when they happen early in development, may cause more detrimental effects than later exposures.

**Fig. 1:**
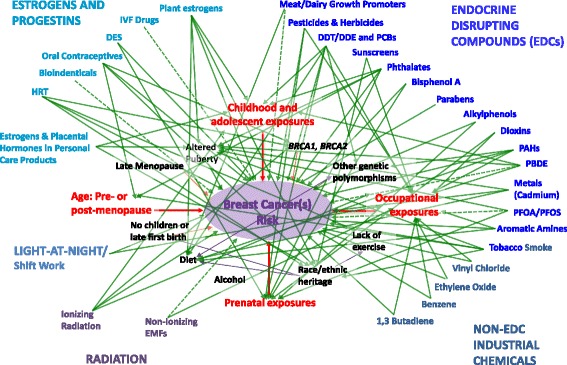
Complexity of factors affecting risk for developing breast cancer. This synopsis of much of the evidence described in this report demonstrates the complexities of the potential connections between exposures to environmental toxicants and development of breast cancer, all embedded in a web-like framework of interconnected factors. *Solid arrows* indicated connections that have been demonstrated directly between exposures and breast cancer risk, or, as appropriate, mediated through factors described in the framing section of this review. These relationships reflect results of the combined human epidemiological and/or animal studies discussed. *Dashed arrows* indicate connections between exposures and risk for breast cancer that are more ambiguous, with evidence coming from non-human or -animal studies, but without the in vivo data to support more directly the link. *Arrows* are not weighted to indicate relative strength of links. Rather the purpose of this model is to demonstrate the complexity of the relationships between environmental factors and breast cancer. (Updated and modified from Gray et al. 2009 [11])

As we move into examining the scientific literature addressing the relationship between various toxicants and breast cancer risk, we offer an interactive model to help situate these data (Fig. 1). While not meant to be fully comprehensive, this model challenges the reader to consider the effects of environmental exposures on disease risk within a complex web-like framework of often interconnected factors, each of which may exert direct, indirect, and interactive effects on cellular processes in mammary tissues [11].

## Evidence linking environmental factors and breast cancer

We turn now to the evidence addressing possible connections between exposures to environmental toxicants and risk for developing breast cancer. In exploring the scientific literature, we draw from relevant human, animal, cell-culture, and high throughput studies. Where possible, we address explicitly the complicating themes raised in the framing section above. And where appropriate, we present conflicting data, especially from the epidemiological literatures, that make clear the nuances of methodology and results that complicate these relationships.

### Hormones: pharmaceutical and personal care products

For decades, scientists have appreciated the positive relationship between lifetime exposures to estrogen and risk for developing breast cancer [122]. More recently it has become clear that long-term exposures to progesterone can also influence the possible development of breast cancer [123]. These exposures are often clumped under the category of ‘reproductive risk factors’ (e.g., age at menarche, menstruation, first full-term pregnancy, and whether or not children were breastfed) in the development of models and simple evaluative tests for determining breast cancer risk [124, 125].

In addition to variations in exposures to endogenous levels of both estrogens and progesterone, there are several other sources of natural and synthetic steroids, including those found in a number of pharmaceuticals and personal care products. Most of these hormonal agents have been designated as carcinogens by the IARC and the NTP (see Table 1). This section examines the relationships between use of these compounds and possible changes in risk for developing breast cancer.

**Table 1:** Carcinogenicity classifications and sources of exposures for hormones in pharmaceuticals and personal care products

Product	IARC	NTP	Source of exposure
Diethylstilbestrol	1	K	Formerly prescribed to pregnant women to sustain viable pregnancies
Hormone Replacement Therapy	1		Treatment of symptoms experienced in menopause
Conjugated equine estrogens	2A		
Medroxyprogesterone acetate			
Bioidentical hormones	1		
Oral contraceptives	1		Contraception
Infertility treatment drugs			Infertility treatment
Clomiphene citrate	1		
Gonadotropins			
Hormones in personal care products	1		Use of placental extracts in personal care products, especially products marketed to women of color

International Agency for Research on Cancer (*IARC*) classifications: 1 = Carcinogenic to humans, 2A = Probably carcinogenic to humans, 2B = Possibly carcinogenic to humans, 3 = Not classifiable as to its carcinogenicity to humans; U.S. National Toxicology Program (*NTP*) classifications: K = Known to be a human carcinogen, RA = Reasonably anticipated to be a human carcinogen. Source of exposure list contains most common exposure sources

#### Diethylstilbestrol

The clearest evidence that a synthetic estrogen can increase risk for breast cancer decades later comes from the tragic experience with diethylstilbestrol (DES). From the 1940s until 1971, doctors prescribed DES for millions of pregnant women to prevent miscarriages and other complications of pregnancy. The drug was banned when daughters of women who took the drug were found to have higher rates of an extremely rare vaginal clear-cell adenosarcoma compared to those who were not exposed to DES in the womb. DES exposure was also associated with an increased risk of breast cancer in the mothers [98, 126, 127].

In a follow-up study of daughters who were exposed prenatally to DES, almost a twofold increase in breast cancer risk was observed in women older than age 40 years (HR = 1.82; 95% CI = 1.04–3.18) [99]. An even greater (three-fold) effect was found for women over the age of 50, although relatively few of the daughters had yet reached that age at the time of the study [101, 102]. Women exposed in utero who had the most severe abnormalities of their vaginal epithelial cells (an indicator of exposures to higher doses of DES) also had a higher risk for developing breast cancer [99].

Studies are just beginning on granddaughters of women prescribed DES during pregnancy, but since these women are only now reaching the ages when breast cancer incidence increases, data sets are too small to reach statistical significance [128]. Relevant rodent models, however, indicate that the F2 generation (granddaughters) of dams exposed to low doses of DES during pregnancy also developed several cancers, including mammary tumors, at rates significantly higher than expected [129].

Studies examining the mechanisms by which DES might be exerting its carcinogenic effects indicate that the compound activates the same subcellular pathways that estradiol does, both by altering cellular metabolism and interaction with DNA [130] and by increasing the rate of breast epithelial cell proliferation [131, 132]. In adult female rats, exposure to DES increased induction of HOTAIR transcription which produces an estrogen-responsive gene silencing protein implicated in the development of breast cancer [133]. DES further dysregulates the expression of estradiol regulated gene expression in adult females, again possibly contributing to an increased risk for breast cancer [134].

Prenatal exposures to DES lead to changes in the adult mammary gland epigenome through alterations in histone methylation, a process that leads to altered gene expression in puberty and adulthood [86, 133, 135]. These epigenetic changes could provide a mechanism for trans-generational effects of DES on breast cancer development [128, 136].

#### Hormone Replacement Therapy (HRT)

The Women’s Health Initiative (WHI) is a large (*n* = 16,608 women) randomized case control study designed to explore the benefits and risks of combined estrogen (conjugated equine estrogens) plus progestin (medroxyprogesterone acetate) HRT in post-menopausal women. In 2002, it was halted after a median follow-up of 5.5 years, three and half years before the intended end of the study period, because researchers observed a significant increase in the relative risk of breast cancer (HR = 1.26; 95% CI = 1.00–1.59) in addition to significant increases in the risk of heart disease, stroke and blood clots [137].

Analyses of a second arm of the WHI study clarified that the increased risk of breast cancer in the WHI study occurred in women taking the combined estrogen-progestin formula, but not for those women taking estrogen-only HRT supplements [138, 139] where a decreased risk for developing breast cancer was found (HR = 0.77; 95% CI = 0.62–0.95). It is critical to note that the estrogen-only option can only be offered to women who have previously undergone surgical hysterectomies because estrogen-only treatment leads to a highly significant increased risk for uterine cancer [140]. One difference between the estrogen-only contraceptives and the combined forms is in the type of estrogen in the formulation. Most often the estrogen in the mixed pill is the semisynthetic compound, ethinyl estradiol, while that in the estrogen only pill is a conjugated equine estrogen. The conjugated form is associated with lower rates of epithelial proliferation in post-menopausal breasts, providing one mechanism by which the two types of interventions might have different effects [141].

Longer-term (median of 13 years) follow-up of both arms of the WHI study indicate that for the women in the combined hormone arm, there was a time dependent and significant increase in risk for developing breast cancer (HR = 0.71; 95% CI = 0.47–1.08 at first year of intervention; 1.36 95% CI = 0.94–1.94 during third year of intervention; 1.65; 95% CI = 1.17–2.32 during the fifth year of intervention). Although there was a sharp decrease in risk after the first year of discontinued use of the mixed HRT formulation, for the full 8-year follow-up period after stopping the hormone treatment, HR values were above 1 (HR = 1.32; 95% CI = 1.08–1.61) [142]. The early, short-term finding is consistent with the rapid drop in post-menopausal breast cancer incidence in the US population since 2002, a decrease that has been attributed to the precipitous drop in HRT prescriptions in selected populations of women (white, middle/upper class, postmenopausal, ER+ tumors) following the release of the data from these large studies [143, 144].

For the estrogen-only arm, the decreased risk of breast cancer remained for the early post intervention phase (HR = 0.55; 95% CI = 73–1.87 for the first 3 years post-intervention) although the benefit disappears over the next 5 years (HR = 1.17; 95% CI = .73–1.87) [142].

Since the results of the original WHI were initially published, other large studies have supported its major conclusions. In 2003, Swedish researchers halted a study of HRT in women with a previous history of breast cancer. Originally planned as a 5-year study, the Swedish trial was stopped after 2 years because women taking combined estrogen-progestin HRT had a significantly increased rate of recurrence or new tumors compared to women who received other treatments for menopausal symptoms ((HR = 3.5; 95% CI = 1.5–8.1) [145].

Also in 2003, researchers in the Million Women Study (MWS) in the United Kingdom reported that the current use of all types of post-menopausal HRT significantly increased the risk of breast cancer (RR = 1.66; 95% CI = 1.58–1.75). Again, the risk was greatest among users of estrogen-progestin combination therapy (RR = 2.00; 95% CI = 1,88–2.12) [146].

Other research has confirmed the basic result that use of combined HRT increases risk of breast cancer in post-menopausal women, and that stopping use of the combination pill leads to decreased risk of developing breast cancer. One study in California found that county-wide decreased incidence in breast cancer was highest (22.6%) in counties with the greatest decline in using HRT, intermediate (13.9%) in counties with moderate decreases in HRT use, and smallest (8.8%) in counties with least decline in HRT use [147].

One study examined breast cancer incidence in BRCA1 mutation carriers who had undergone oophorectomy to prevent onset of ovarian cancer. Short-term use (median = 4.27 years) of HRT was not associated with any change in risk of developing breast cancer (OR = 0.80; 95% CI = 0.55–1.16), regardless of HRT formulation (estrogen alone or estrogen + progestin) [148].

Another study examining the possible interactions between use of HRT and race, weight, and breast density found that HRT use increased risk for breast cancer in white (OR = 1.21; 95% CI = 1.14–1.28), Asian (OR = 1.58; 95% CI = 1.18–2.11) and Hispanic (OR = 1.35; 95% CI = 1.09–1.67) women, but not Black women (OR = 0.91; 95% CI = 0.72–1.14). There was no interaction between HRT use and either BMI or breast density [149].

A meta-analysis that included 116,304 breast cancer cases demonstrated that women who engage in high levels of physical activity have a significantly reduced risk of developing breast cancer (SRR = 0.88; 95% CI = 0.85–0.90), with decreases being found in both ER+/PR+ and ER−/PR− cancers. However, women who used HRT had no decrease in breast cancer risk when they engaged in vigorous physical exercise [150].

Examination of cancer histology in women taking combined HRT at the time of diagnosis reveals an increased presentation of breast cancer of lobular origin [151–153], but also of cancers with low proliferation rates (mitotic indices) and favorable prognostic outcome [153, 154].

#### Bioidentical hormones

Following the results of the major studies implicating HRT as being causally related to postmenopausal breast cancer, many women turned to alternative sources of hormone therapy to treat their menopausal symptoms with hopes of finding safer options. For many women, this meant using ‘bioidentical hormones’ of some sort, hoping to mimic the effects of natural hormones without succumbing to the negative health outcomes associated with traditional HRT [155]. Unfortunately there have been very few studies examining the relationship between taking bioidentical hormones and later development of breast cancer. Perhaps more importantly, and confusing the conversation on this topic, the term ‘bioidentical hormones’ is used in many different ways with potentially different implications for associations with health outcomes [156]. The most conservative definition, adopted by the Endocrine Society, is for compounds that ‘have exactly the same chemical and molecular structure as hormones that are produced in the human body’ [156]. Bioidentical hormones may be synthesized or derived from plant sources.

A few types of bioidentical hormone composites or individual components have been tested and approved by the Food and Drug Administration (FDA). But the increasingly common use of individually compounded bioidentical hormone regimens has not been tested for safety or associated health outcomes and the consistency of prescribing and providing individualized compounded formulae varies enormously [156, 157].

The strongest evidence for a lack of association between use of bioidentical hormones and possible development of breast cancer comes from data examining the use of the natural hormone progesterone, instead of MPA or other synthetic progestins, as part of the HRT regime [158]. Research indicates that increased exposure to natural progesterone did not increase risk for breast cancer and, in some circumstances, might even be protective [159, 160]. In the single large-scale cohort study examining risks for breast cancer in women taking hormone replacement regimens with either natural progesterone or synthetic progestins compounded with estrogens, use of a progesterone-based replacement was associated with no added risk for breast cancer compared with controls (RR = 1.00; 95% CI = 0.83–1.22), while women who took combined HRTs that included synthetic progestins had significantly increased risk for developing the disease (RR = 1.69; 95% CI = 1.50–1.91) [161]. This difference was particularly prevalent in the incidence of ER+ tumors, especially ER+/PR− masses (RR = 2.6; 95% CI = 1.9–3.5) [162].

Less positive news comes from a study comparing the effects of conjugated equine estrogens, the major estrogenic component in traditional combined estrogen-progestin HRT, with natural estradiol in a primate model of postmenopausal breast cancer. In this study, natural estradiol induced greater proliferation of breast epithelial cells than did the conjugated form [141].

#### Oral contraceptives

Numerous studies have demonstrated an increased risk of breast cancer in women using oral contraceptives. The risk for breast cancer is greatest among current and recent users of oral contraceptives, particularly those who have used them for more than 5 years and initiated use at a young age [163–168]. For example, in a large prospective cohort-study, an increased incidence of breast cancer was found in women who were younger than age 50 at the time of diagnosis and had begun use of oral contraceptives before the age of 20 — as compared with those who started later (HR = 3.26; 95% CI = 1.06–10.01). Women who had begun use before the age of 20 and were older than age 50 at the time of diagnosis showed no increased risk compared to age similar cases who began use later (HR = 0.70; 95% CI = 0.33–1.46). Women in this study took contraceptives for an average of 6 years, although the duration of use varied from 2 ½ to 12 years [169].

Sweeney et al. examined possible effects of oral contraceptive use on later risk for breast cancer in Hispanic and non-Hispanic white women. Statistically, Hispanic women have somewhat lower rates of breast cancer than do white women and they are more likely to have ER− tumors. However, use of oral contraceptives during the previous 5 years led to significant increases in breast cancer incidence in both groups. The effect was magnified for women of both groups when oral contraceptive use continued for more than 20 years (OR = 2.23; 95% CI = 1.17–4.25 for ER− tumors). Mirroring other study evidence, and again for both Hispanic and non-Hispanic white women, significant increases in ER+ tumors were observed [170].

Researchers in the Black Women’s Health Study, a large (over 53,000 women) prospective study of women across the U.S., report that use of oral contraceptives by African American women was associated with a higher risk of receptor negative (ER−, PR−) cancer than women who did not use the pill (IRR = 1.65; 95% CI = 1.19–2.30). The risk for later diagnosis of ER−/PR− breast cancer increased as the duration of contraceptive use was prolonged among women who took the pill and were still using it within the past 5 years (trend *p* = 0.001). The only significant effect of oral contraceptive use on development of ER+/PR+ cancers in this cohort was for women who had taken the pill for more than 10 years (IRR = 1.45; 95% CI = 1.02–2.07) [171].

Women with *BRCA1* or *BRCA2* mutations, as well as women with family histories of breast or ovarian cancer, have an increased susceptibility to the risk-inducing effects of oral contraceptive usage [166, 172, 173]. Paternal contribution (as compared to maternal contribution) of the *BRCA* mutation confers greater risk for women with this genetic variation who also use oral contraceptives (HR = 1.84; 95% CI = 1.46–2.34) [174]. One mechanism by which the interaction between *BRCA* gene status and use of oral contraceptives may influence breast cancer risk, is by altering the sensitivity and activity of progesterone in breast cancer cells, both by increasing the synthesis of PR in the cells and by enhancing the responsiveness of progesterone-regulated genes [175].

Use of oral contraceptives is associated with an increase in later-stage (type II or greater) breast tumors [176], tumors originating in the lobular tissue [171], as well as with the ER− profile of the disease [171, 177]. Significant associations between use of oral contraceptives and development of the aggressive triple negative (ER−/PR−/Her-2R-) form of the disease was found in a primarily White cohort (OR = 2.5; 95% CI = 1.4–4.3) [178] as well as in a cohort of African American women (OR = 1.78; 95% CI = 1.25–2.53) [179]. Use of oral contraceptives for 10 or more years has also been associated with a diagnosis of comedo DCIS (OR = 1.31; 95% CI = 0.70–2.47) [180], the most aggressive form of DCIS which is sometimes confused with early forms of invasive breast cancer [181].

Post-menopausal women who used oral contraceptives for eight or more years, but who have discontinued use for at least a decade, show no significant increase in breast cancer rates [182, 183].

Two studies have examined the relationship between use of injectable progestin-only contraceptives and breast cancer incidence. Both studies found increases in breast cancer risk that were significant, but rates decreased to normal within a few years after stopping use of the drugs [184, 185].

#### Infertility treatment drugs

Despite the substantial evidence linking HRT and oral contraceptive use with increased incidence of breast cancer, neither the condition of subfertility nor the use of infertility-treatment (ovulation-stimulation) drugs appears to have a clear link to the disease [186–189]. This is true also when the study involves infertile women who are also *BRCA* carriers [190]. Where a link has been found, it has been for women who gave birth to more than one infant as a result of their IVF treatment (HR = 1.44; 95% CI = 1.06–1.97) [191] and those who have been treated with high doses of clomiphene citrate.

Two studies found increased risk of breast cancer for women who have been treated for ovarian infertility with drugs including gonadotropins or clomiphene citrate. However, the results were significant only when the incidence of breast cancer was compared with the general population of women, but not with the more appropriate control of women with ovarian infertility who have not been treated with fertility drugs [192, 193]. Two other studies, however, have found statistically significant increases in breast cancer rates in women taking clomiphene citrate compared with rates for infertile women taking no infertility treatment (HR = 1.42; 95% CI = 0.99–2.55) [194]; (OR = 2.7; 95% CI = 1.3–5.7] [195]. A smaller subgroup of women whose infertility was not ovarian in origin and who underwent multiple treatments with high doses of clomiphene citrate, had increased risk of later developing breast cancer compared with women in the general population (OR = 3.0; 95% CI = 1.35–6.67) [188].

Another study complicates the story, however. Within the cohort of women with fertility problems, there was no difference in the rate of breast cancer when general comparisons were made between women who had taken fertility drugs and those who had not. But when age of treatment was factored in, a significant increase in risk for breast cancer was found in women who had begun infertility drug treatments before the age of 24, as compared with infertile women of the same age who had not undergone drug IVF and associated drug treatments (HR = 1.59; 95% CI = .1.05–2.42). Increased risks for breast cancer were not associated with infertility treatment in older women (after aged 40 years) who underwent IVF protocols [196]. These data are consistent with a model in which younger adult breast cells are more sensitive to the perturbations and/or protections resulting from altered exposures to both endogenous and exogenous sources of hormones.

#### Hormones in personal care products

Placental extracts, probably with high concentrations of progesterone [197] and estrogenic chemicals [198] are sometimes used in cosmetics and hair care products, particularly products marketed to women of color. Addition of hormones and extracts is advertised to promote growth and thickness of hair. However, research indicates that use of these products in infants and children may also be linked to precocious puberty or early sexual maturation [191, 199, 200], a risk factor for later life breast cancer [201]. Scientists have proposed that use of these hormone-altered products might be contributing to the increased incidence of breast cancer, especially among young African American women who use these products more than their white counterparts [202, 203].

Seven of eight extracts from skin and hair products commonly used by African American women had effects on proliferation of MCF-7 cells in culture; four of the seven were estrogenic while three showed antiestrogenic activity [204].

Hormones, especially estrogens, are also regularly added to anti-aging creams [205], because of their effectiveness in raising collagen count, as well as skin hydration. Together, these two factors are thought to decrease wrinkling of the skin [206], but they can also increase women’s total lifetime exposure to estrogen.

*Section summary:* There is clear evidence that exposure to DES during gestation increases the risk for developing breast cancer in the women who were exposed in utero, and also for their mothers and possibly their daughters. Post-menopausal use of HRT compounded with synthetic estrogens and progestins also increases the likelihood of developing breast cancer although use of estrogen-only HRT has protective effects for those women who have undergone a hysterectomy. Compounding HRT drugs with the natural hormone, progesterone, does not appear to have detrimental effects on breast cancer risk, although use of the natural estrogen, estradiol, may increase breast cell proliferation and consequent risk for developing breast cancer. There is little consistent evidence that use of hormonal drugs in IVF procedures alters risk for breast cancer, although there are numerous methodological issues in these studies. Finally, several personal care products, especially those marketed primarily to communities of color, have estrogenic and progestin additives, increasing lifetime exposures to these hormones.

### Endocrine disrupting compounds (EDCs)

Although intentional use of natural and synthetic hormones has been a practice for decades, if not centuries, it is only in the past two decades that scientists have come to recognize that many common products also contain chemicals that are disruptive to the exquisitely sensitive endocrine system [207]. These chemicals, found in products as different as plastics, pesticides, fire retardants, and sunscreen, were added to the manufactured products for reasons not intentionally related to their endocrine-related properties. Nevertheless, many compounds have been shown to fit the Endocrine Society’s definition of an endocrine disrupting compound (EDC), “an exogenous chemical, or mixture of chemicals, that interferes with any aspect of hormone action” [47].

By interfering with the actions of natural hormones, exposures to EDCs have been shown to contribute to the development of a wide variety of disease states [49, 51]. Often these effects are most profound when exposures are low-dose [38] and during early development [48]. This section addresses the growing literature on the connections between several important EDCs and the risk of developing breast cancer, mainly – but not exclusively – from non-human models. Although we mostly treat the chemicals independently, as is true of the research literature, we recognize the importance of exposures to mixtures of EDCs as these substances infuse the products we use, and also the air we breathe, the water we drink, and the surfaces on which we work and play. While most of these EDCs have not been formally evaluated for carcinogenicity, Table 2 demonstrates the almost ubiquitous presence of these chemicals in our environment.

**Table 2:** Carcinogenicity classifications and sources of exposures for endocrine-disrupting compounds (EDCs)

EDC	IARC	NTP	Sources of exposure
Bisphenol A			Polycarbonate plastic, epoxy resins linked food cans, dental sealants, thermal receipts
Phthalates			Fragrance ingredients in personal care and cleaning products, plastics. Also pharmaceuticals, building materials, insecticides and food packaging/food processing.
di(2-ethylhexyl)phthalate (DEHP)	2B	RA	
di-*n*-butylphthalate (DNP/DBP)			
monoethyl phthalate (MEHP)			
diethyl phthalate (DEP)			
butyl benzyl phthalate, (BBP)	3		
di-*n*-octyl phthalate, (DOP)			
di*-i-*butyl phthalate (DiBP)			
monomethyl phthalate			
Parabens			Antimicrobial preservatives in food, personal care products, soaps and detergents, and pharmaceuticals
methyl-paraben			
propyl-parabens			
butyl-parabens			
Alkylpenols			Detergents and cleaning products, antioxidants in plastic and rubber products
4-nonylphenol (4-NP)			
4-octylphenol (4-OP)			
Triclosan & Triclocarban			Antimicrobials in liquid hand soap, other personal care products and household items
EDCs found in sunscreens			UV filters
3-(4-methylbenzylidene)-camphor (4-MBC)			
octyl-methoxycinnamate			
octyl-dimethyl-PABA (OD-PABA)			
benzophenone-3 (Bp-3)			
homosalate (HMS)			
Perfluorooctanoic Acid (PFOA) & Perfluorooctanoic Sulfate (PFOS)	2B		Stain resistant coatings, non-stick coatings, commercial products including firefighting foams.
Polycyclic Aromatic Hydrocarbons (PAHs)		RA	Byproducts of combustion resulting from fossil fuel production, diesel exhaust, grilled meats, cigarettes.
Pyrene			
benz[a]anthracene	2B	RA	
benzo[a]pyrene	1	RA	
Triazine herbicides			Weed control for corn and sorghum crops.
Atrazine			
Simazine			
Cyanazine			
Other Pesticides & Herbicides			
Heptachlor	2B		Insecticide, now banned
Dieldrin and Aldrin	2A		Insecticide for corn and cotton, now banned
Chlordane	2B		Home termites, general crop pesticide
Malathion	2A		Residential, recreational, crop pesticide
2,4-D	2B		Broadleaf weed herbicide
2,4,5-trichlorophenoxypropionic acid (2,4,5-TP)			Woody plant and broadleaf weed herbicide, now banned
Persistent organochlorines			
Dichloro-diphenyl-trichloroethane (DDT)/DDE	2A	RA	Insecticide, now banned
PCBs	1	RA	Electrical insulation, fluid coolants, plasticizer in paints, dyes & inks
Dioxins: 2,3,7,8-tetra chlorodibenzo-para-dioxin (TCDD)	1		Byproduct of burning of chlorine-based chemicals
Polybrominated Diphenyl Ether (PBDEs)			Flame retardants, previously used in furniture and electronics; most have been banned or voluntarily phased out
Aromatic amines			
o-toluidine	1	K	Hair dyes
4-aminobiphenyl (ABP)	1	K	Azo dyes in textiles
p-phenylenediamine			Hair dyes
2-amino-phenylimidazo[4,5-b]pyridine (PhIP)			Cooked meats
heterocyclic aromatic amines			Hair dyes
Metals			Naturally occurring elements; contaminants in naturally derived colorants, clays, and other metals, found in cosmetics, toys, and other products.
Copper			
Cobalt	PO	RA	
Nickel	PO		
Lead	2B	RA	
Mercury			
Methylmercury	2B		
Tin			
Cadmium	1	K	
Zinc			
Iron	1		

International Agency for Research on Cancer (*IARC*) classifications: 1 = Carcinogenic to humans, 2A = Probably carcinogenic to humans, 2B = Possibly carcinogenic to humans, 3 = Not classifiable as to its carcinogenicity to humans; U.S. National Toxicology Program (*NTP*) classifications: K = Known to be a human carcinogen, RA = Reasonably anticipated to be a human carcinogen. Source of exposure list contains most common exposure sources

#### Bisphenol A (BPA)

The ubiquitous synthetic chemical bisphenol A (BPA) is the main component used in the manufacturing of polycarbonate plastic and is found in many common household products. It is also found in dental sealants, thermal receipts, food packaging, and epoxy resins lining food cans. Significant levels of BPA have been measured in ambient air [208], house dust [209], river and drinking water [210].

BPA is an unstable, lipophilic compound that can leach into food products, especially when heated [211], and a major source of exposure to BPA is thought to be through food products contaminated with the chemical [212, 213]. Two studies have explored the effects of increased ingestion of food and drink packaged in materials containing BPA. Both found rapid increases in BPA levels in urine and/or blood samples taken from subjects who intentionally increased their intake of common foods and drinks packaged in BPA-containing products [214, 215]. Another study took the opposite approach and demonstrated that just a 3-day period of limiting intake of packaged foods decreased the concentrations of BPA found in urine by an average 65% [216].

Samples taken from fasting people indicates that sources other than foods may also be responsible for the pervasive exposure to BPA, as levels of the chemical did not decrease as rapidly as would have been predicted were food the only source of contamination [217]. Of growing concern are the high levels of BPA that are transferred to our skin and then rapidly absorbed by holding BPA-containing thermal receipts [218].

Clearance of BPA from the body is quite rapid, with its urinary half-life on the order of hours to days [217]. Despite its rapid clearance rate, BPA was found in 93% of about 2500 urine samples from a broad national sample of adults through the NHANES study [219]. BPA has been found in the blood and urine of pregnant women [220–222], and in breast milk soon after women gave birth [223, 224]. BPA has also been found in blood samples from developing fetuses and the surrounding amniotic fluid [225]; in placental tissue and umbilical cord blood at birth [226, 227]; and in the urine of premature infants housed in neonatal ICUs [228].

Many studies using both rat and mouse models have demonstrated that even brief exposures to environmentally relevant doses of BPA during gestation or around the time of birth lead to changes in mammary tissue structure predictive of later development of tumors [90, 229, 230]. Early exposure to BPA led to abnormalities in mammary tissue development that were observable during gestation and were maintained into adulthood [92, 231, 232]. Many of these changes are similar to those observed after prenatal exposure to DES [132]. Prenatal exposure of rats to BPA resulted in increases in the number of pre-cancerous lesions and in situ carcinomas [233, 234], as well as an increased number of mammary tumors following adult exposures to sub-threshold doses of known carcinogens [235, 236] or without the addition of the additional carcinogen [234].

Prenatal exposure to BPA changes the gene transcription in both the epithelial and stromal compartments of the mouse fetal gland, through mechanisms that are mediated through both ER-dependent and ER-independent pathways [237, 238]. Both BPA and DES exposures alter the expression of several genes involved in extracellular matrix formation, as well as adipogenesis and lumen formation [237]. BPA acts on estrogen-independent pathways to alter the epithelial-mesenchymal transition (EMT) via down-regulation of FOXA1, a key regulator of hormone responses in breast cancer cells [238]. These data suggest that during gestation, BPA acts on stromal cells to alter the collagen fiber content and expression of several proteins including receptors mediating signaling pathways, which then alter epithelial gene expression and cell proliferation [237, 239].

Neonatal exposure of mice to BPA increased sensitivity to estradiol-mediated development of mammary gland structures at puberty [240] as well as increased synthesis of the progesterone receptor and activation of progesterone-regulated mammary-cell proliferation [132].

Changes in mammary development comparable to those observed in rodent models were also observed when female rhesus monkeys were exposed to environmentally relevant doses of BPA during gestation [241].

Some of the long-term effects of neonatal exposures to BPA may be dose dependent, with low- and high-dose exposures resulting in different timing and profiles of changes in gene expression in cells of the mammary gland. In one study, low-dose exposures had the most profound effect on rat mammary glands during the period just prior to the animals’ reaching reproductive maturity, while higher doses had more delayed effects, altering gene expression in mammary tissues from mature adults [55]. Prenatal exposure to low doses of BPA altered mammary gland development in adult rats, while higher doses did not [56]. In a study of chronic exposure of adult mice to different concentrations of BPA, only low doses decreased the latency of tumor appearance and increased the number of mammary tumors as well as their rate of metastasis. All doses enhanced the rate of mammary cell proliferation, but only relatively higher doses counteracted this increased proliferation with parallel increases in programmed cell death [242]. And in an evaluation of prenatal exposures to BPA in male rats, non-linear dose-response effects of BPA were found for development of mammary gland structures [243].

In addition to physical abnormalities in the developing mammary tissue of rodents treated perinatally with low levels of BPA, there are also functional deficits. Female rats exposed to BPA during gestation and suckling had physical abnormalities in their adult mammary tissue as well as decreases in yield and altered protein content of their own milk when, as new mothers, they were feeding their pups. Observed differences following BPA exposure were similar to those found in rats that had been similarly exposed to DES, a known breast carcinogen [244].

Studies using cultures of human breast cancer cells demonstrate that BPA acts, in part, through the same cellular response pathways as the natural estrogen estradiol [245, 246]. BPA binds weakly to the intracellular ER, and also affects cellular functions through interactions with the membrane ER (mER) [247, 248]. But BPA also exerts disruptive effects on cell processes, including changes in activation of signal transduction pathways in ER− cell lines [238, 249]. Beyond binding to ER, BPA binds to the orphan nuclear receptor estrogen-related receptor gamma (ERRγ), a protein to which estradiol does not bind [250–253]. The nuclear receptor family is involved in a wide scope of biological processes, from embryonic development and differentiation through normal maintenance of homeostatic systems to the dysregulation of these processes involved in the development of cancers [254]. BPA also binds to both the androgen receptor (AR) and the thyroid hormone receptor (TR) [255–257], altering activities of those hormone-regulated systems.

Prenatal exposure of mice to BPA also resulted in dysregulation of inflammatory cytokines in adult mammary tissue, a process that may lead to altered cell growth through inhibition of immune responses that commonly target developing cancer cells [258].

Exposure of normal and cancerous human breast cells to low levels of BPA led to altered expression of hundreds of genes including many involved in hormone-receptor-mediated processes, cell proliferation and apoptosis, and carcinogenesis [259–261]. In the presence of BPA, cells derived from the non-cancerous breast of women diagnosed with breast cancer had a gene-response profile associated with the development of highly aggressive tumors [262].

Effects of BPA on mammary tissue development may also be manifested via epigenetic mechanisms, leading to changes in gene regulation across the lifetime. Prenatal exposures of rats to low levels of BPA altered the epigenome in mammary tissue with different profiles being observed at weaning and post-puberty [135]. Exposures to either BPA or DES lead to similar changes in the adult mammary gland epigenome through alterations in histone methylation and gene silencing, processes that lead to altered gene expression in puberty and adulthood [86, 133–135].

BPA reduces the efficacy of common chemotherapy agents (cisplatin, doxirubicin and vinblastin) in their blocking the proliferation of human breast cancer cells when tested in vitro [263, 264].

#### Phthalates

Phthalates are a group of endocrine-disrupting chemicals commonly used to render plastics soft and flexible. They are found in a wide variety of common products including plastics (e.g., children’s toys), cosmetics, pharmaceuticals, baby care products, building materials, modeling clay, automobiles, cleaning materials and insecticides [265]. Phthalates are readily absorbed through the skin [266] and can also enter the body through inhalation or medical injection procedures [267]. Other major sources of at least one phthalate, di(2-ethylhexyl)phthalate (DEHP), are food packaging [268, 269] and fast food [270]. A dietary intervention study has demonstrated that just a 3-day period of limiting intake of packaged foods decreased by half the concentrations of DEHP found in urine [216]. Another dietary intervention in which study participants followed a 5-day monastic lifestyle, including a vegetarian diet, led to a significant decrease in urinary phthalate levels [271]. Significant levels of DEHP and another phthalate used in food packaging, di-*n*-butylphthalate (DNP), were found in cooked foods, both before and after packaging, that were served to children through school meal programs [272]. Many wines and liquors, as well as spices, are contaminated with phthalates resulting from leakage of the chemicals from storage containers [273, 274].

Phthalates have been found in indoor air and dust [275] and in human urine and blood samples from children, adolescents, and adults [216, 276–278], as well as in amniotic fluid from pregnant women [279]. Phthalates have also been detected in human breast milk and urine [280, 281]. Phthalates cross the human placenta, exposing fetuses to the hazards associated with exposure to an important class of EDCs during this critical period of development [282]. Young infants are also exposed to high levels of phthalates, with measurable levels of seven different phthalates being found in infants born between 2000 and 2005 [283].

A 2012 study examined whether or not there is a relationship between urinary levels of nine different phthalates and incidence of breast cancer. In this study, urinary phthalate metabolites were detected in 82% of the women, whether or not they had been diagnosed with breast cancer. Elevated levels of monoethyl phthalate (MEP), a urinary metabolite of the parent compound diethyl phthalate (DEP; often used in fragrance), was associated with increased risk of breast cancer (OR = 2.20; 95% CI = 1.33–3.63). This association was highest in premenopausal women (OR = 4.13; 95% CI = 1.60–10.70). Metabolites of two other common phthalates (butyl benzyl phthalate, BBP, and di-*n*-octyl phthalate, DOP) were negatively associated with breast cancer risk in this study (BBP: OR = 0.46; 95% CI = 0.27–0.79 and DOP: OR = 0.44; 95% CI = 0.24–0.80) [284]. Higher levels of urinary mono(2-ethylhexyl)phthalate (MEHP), a marker of DEP body burden, have also been associated with increased pregnancy loss in a study of Danish women [285].

Phthalates are considered to be endocrine disruptors because of their complex effects on several hormonal systems including the estrogen and androgen hormone systems. Some phthalates, including BBP and DBP, act as weak estrogens in cell culture systems. They can bind to estrogen receptors (ER), induce estrogen-appropriate cellular responses and act additively with estradiol in altering these systems [286, 287]. DBP, di*-i-*butyl phthalate (DiBP) and BBP also bind weakly to the androgen receptor (AR), disrupting the cellular actions ordinarily initiated by the androgens [288, 289]. In breast cancer cell lines, BBP promotes cancer stem cell growth through activation of the aryl hydrocarbon receptor (AhR) [290]. Phthalates can also induce proliferation, malignant invasion, and tumor formation in breast cancer cell lines that are receptor negative, indicating that at least some effects of these compounds are independent of their direct estrogenic or androgenic effects [291, 292].

The endocrine-disrupting properties of this class of chemicals have been well established in the offspring of mother rats who had been treated with phthalates while pregnant. Phthalates disrupt the development and functioning of male and female reproductive systems by interfering with the production of testosterone and estradiol, respectively [293, 294]. Abnormalities in male offspring exposed prenatally included nipple retention, shortened ano-genital distance and increased cryptorchidism [295, 296]. Exposure of human mothers to phthalates, as measured by analysis of their urine samples, has also been associated with shortened ano-genital distances in their newborn sons — a measure of feminization of external genitalia [297, 298].

A case-control study examined phthalate levels in apparently healthy girls who went through thelarche (breast development) before the age of 8, as compared with girls who underwent precocious puberty because of abnormalities in their neuroendocrine systems and with girls who were progressing through puberty at normal ages. Increased levels of monomethyl phthalate (MMP) were associated with early thelarche group, but not either of the comparison groups [299]. Early breast development in otherwise healthy girls is associated with an increased risk for breast cancer [300].

Exposure of very young rats to BBP resulted in increased cellular proliferation in the terminal end buds of mammary tissue. BBP-induced changes in mammary cell gene expression profile were consistent with abnormalities in cellular differentiation and cell-cell communication [301]. Similar structural irregularities were observed in post-natal development of mammary tissues when rats were exposed to the BBP only in utero when their mothers were fed low levels of the compound during the second half of their pregnancies [302].

DEHP has been shown to alter cellular mechanisms at a number of different levels, including inducing damage to DNA leading to altered rates of mitosis and apoptosis; increases in cell proliferation, tumor cell mobility and invasiveness; and decreased intercellular communication at gap junctions. DEHP also enhances the transition of epithelial cells to mesenchymal cells, thus gaining both migratory and invasive potentials [303]. Exposure of normal human breast epithelial cells to DBP resulted in changes in gene expression in pathways related to a number of systems, including immune responses, cell cycle regulation and antioxidant status of the cell [304].

BBP, DBP and DEHP all significantly increased cell proliferation in MCF-7 breast cancer cells. In addition, these three phthalates inhibited the anti-tumor action of tamoxifen in MCF-7 breast cancer cells [305]. BBP also decreased the efficacy of the chemotherapeutic agents, doxorubicin and cyclophosphamide [306].

#### Parabens

Parabens are a group of compounds widely used as antimicrobial preservatives in food, pharmaceutical and cosmetics products. Parabens are absorbed through intact skin and from the gastrointestinal tract and blood. Parabens have been found in almost all urine samples examined from a demographically diverse sample of U.S. adults through the National Health and Nutrition Examination Survey (NHANES) study. Adolescents and adult females had higher levels of urinary methyl paraben and propyl paraben than did similarly aged males [307]. Parabens are also found in amniotic samples during the second trimester of pregnancy [308].

Measurable concentrations of six different parabens have been identified in biopsy samples from breast tumors [309]. The particular parabens were found in relative concentrations that closely parallel their use in the synthesis of cosmetic products [310]. Higher levels of n-propylparaben were found in the axilla quadrant of the breast [311], the region in which the highest proportion of breast tumors are found, although concentrations were not related to the actual location of tumors in breasts of individual women. Several investigators have noted the importance of studying the effects of mixtures of parabens, in concentration profiles that are relevant to natural exposures to the compounds, to understand the complex effects of this class of chemicals on increased risk for developing breast cancer [312–314].

Parabens are weak estrogen mimickers, with the potency of the agonistic response being related to the alkyl side group structure [315, 316]. They can bind to both ERα and ERβ, with higher affinity to the ERβ site [316], and they increase the expression of several estrogen-responsive genes involved in cell growth and proliferation as well as inhibition of apoptosis [317–320]. Adding paraben mixtures at concentrations and combinations measured in breast biopsy tissue led to increases in MCF-7 growth and proliferation [321].

Methyl-, propyl- and butyl-parabens all stimulated proliferation in ER+ human breast cancer (MCF-7) cells, as well as in non-malignant human breast epithelial (MCF-10A) cells. The parabens increased estrogen secretion in the MCF-7 cells, but decreased it in the MCF-10A cells [321]. Follow-up work demonstrated that the proliferative effect of parabens on MCF-7 cells was independent of direct effects on either cell cycle or apoptosis gene expression. On the other hand, in the MCF-10A cells, the parabens mimicked estradiol in altering expression of genes involved in both cell cycle progression and apoptosis [322].

When added together to cultures of ERα- and HER2-positive human BT-474 breast cancer cells, butylparaben and heregulin, a natural HER ligand, led to a synergistic increase in the oncogene *Myc* mRNA expression and cell activity. These data indicate ligands for the two receptors can engage in crosstalk in breast cancer cells, increasing the effects of exposures to environmental parabens [323]. At concentrations lower than those found in breast cancer samples, parabens also exerted inverse antagonistic effects, thereby mimicking the effects of estrogenic stimulation, at the membrane ERR_Y_ [324].

Seventeen days treatment of nonmalignant human breast (MCF-10A) cells with methyl-, propyl-, or butyl-parabens led to induction of a transformed phenotype linked to the process of breast cell carcinogenesis [325]. Longer-term (>20 weeks) treatment of MCF-7 cells with the same parabens at the concentration that leads to maximal increase in cell proliferation enhanced migratory and invasive responses [326].

In breast epithelial cells derived from women at high risk for developing breast cancer, methylparaben countered the apoptotic effect of tamoxifen, a major adjuvant treatment of breast cancer [318].

#### Alkylphenols

Alkylphenols are industrial chemicals used in the production of detergents and other cleaning products, and as antioxidants in products made from plastics and rubber. They are also found in personal care products, especially hair products, and as an active component in many spermicides. In the Silent Spring Institute study of household contaminants, alkylphenols—especially 4-nonylphenol (4-NP) and its breakdown products—were found in all samples of house air and 80% of house dust samples [202]. Substantial concentrations of these chemicals have also been found in wastewater associated with domestic greywater and sewers, urban wastewater and municipal landfills [327–330].

NHANES data examining chemical levels in urine of American adults found 4-NP in 51% of samples evaluated [331] and 4-octylphenol (4-OP) in 57.4% of samples [219]. Similar results were found in serum samples of nursing Swedish women 3 weeks after they had given birth [332], and significant levels of both 4-NP and 4-OP were found in breast milk samples from Taiwanese women [333].

Alkylphenols, including 4-NP, have been shown to mimic the actions of estradiol, mediating their effects through the cellular estrogen receptor. They also bind to the cell membrane ER and mimic cellular signaling responses usually controlled by estradiol [334]. In a study examining the effects of 4-NP in human breast tumor cells (MCF-7) in vitro, changes in gene expression were observed in several genes involved in cell proliferation, DNA transcription and cell signaling—all systems that are disrupted in tumor formation [335–337].

Prenatal exposure of rats to 4-NP caused altered development of the mammary gland as well as changes in steroid-receptor populations in several reproductive tissues [338]. Treatment of mice with 4-NP led to an increased synthesis of estriol, a weak natural estrogen, by the livers of the treated animals. When compared with mice treated with equivalent amounts of estradiol, the mice exposed to 4-NP had an increased risk of mammary cancer [339].

#### Triclsoan and triclocarban

Triclosan and triclocarban are antimicrobial agents that have been used broadly in a wide range of personal care, household and industrial products over the past 40 years [340]. The chemical structure of triclosan has similarities to both thyroid hormone (T_4_) as well as several known endocrine disruptors including PCBs, DES and bisphenol A, while triclocarban has similar chemical properties to several pesticides and pharmaceuticals [341]. Both are found in freshwater samples, especially in lakes and downstream from wastewater treatment plants, in concentrations known to be harmful to wildlife [342–344].

In a study of adult American urine samples as part of the CDC NHANES study protocol, 75% of samples were found to have significant levels of triclosan and its metabolites. Higher levels were found in younger and more affluent adults [34]. A 10-year trend analysis of NHANES urinary triclosan levels found a small decrease in levels in the 6 years since they peaked in 2006. A parallel NHANES study examining chemical levels in pregnant women found measureable levels of triclosan in 87% of urine samples examined [345]. In a smaller study of American adult samples, triclocarban and its metabolites were detected in one third of urine and one half of serum samples that were tested [346]. Human autopsy analysis reveals that triclosan bioaccumulates in liver and adipose tissue, but not brain, the three tissues examined [347].

Although there has been very little work examining the direct effects of either triclosan or triclocarban on mammary system development or risk for developing breast cancer [348], considerable research demonstrates that these two compounds exert effects on hormonal systems in ways similar to established mechanisms for perturbing normal breast development and health. Depending on the concentration of chemical tested and the model system used, triclosan and triclocarban exert a complex combination of estrogenic and antiestrogenic, and androgenic and antiandrogenic effects, all mediated at least in part through interactions with the estrogen receptor (ER) and androgen receptor (AR) of target cells [340–342, 350, 351].

At even a very low dose, triclosan was estrogen-like in enhancing proliferation rates of cultured human breast cancer cells (MCF-7 BOS line), yet in combination with low doses of estradiol, triclosan was antiestrogenic in suppressing the full estradiol-induced increase in cell growth and proliferation. At higher, but still environmentally relevant concentrations, triclosan decreased the viability of the cells [351]. Also in MCF-7 cells, triclosan significantly enhanced both cyclin D1 and D2 activity and increased cell proliferation. These effects were blocked by concurrent treatment with ICI-182,780, a specific ER antagonist [352].

In addition to its effects exerted through the steroid receptor systems, triclosan has been shown to alter levels of thyroid hormone in pubertal rats [353, 354]. Treatment of mother rats with triclosan during the period of lactation led to a sustained 3-week decrease in thyroid hormone in the dams. However, pups, only had suppressed T4 levels for the first few days of suckling, with normal levels being recorded later in the period despite continued exposure to maternally ingested triclosan [355].

#### Hormonally active chemicals found in sunscreens (UV filters)

Concern about exposure to ultraviolet (UV) radiation from the sun and the risk of skin cancer has led to widespread use of sunscreens. Many sunscreens contain chemicals that are not only estrogenic but also lipophilic. Studies show these chemicals are accumulating in wildlife [356], and are found in human urine and breast milk samples [357, 358]. NHANES data indicate that over 96% of American adults have detectable levels of benzophenone-3 (Bp-3) in their urine [219], and that urinary levels have increased in the years between 2006 and 2012. Levels were higher in women and non-Hispanic whites than in other groups [351]. A recent occupational study of firefighters found that their Bp-3 levels were five times those reported in the NHANES studies [359].

Of six common sunscreen chemicals, five of them exerted significant estrogenic activity as measured by increased proliferation rates of human breast cancer cells (MCF-7 cells) grown in vitro. These chemicals were 3-(4-methylbenzylidene)-camphor (4-MBC), octyl-methoxycinnamate (OMC), octyl-dimethyl-PABA (OD-PABA), benzophenone-3 (Bp-3) and homosalate (HMS) [360, 361]. The results for 4-MBC have been replicated in another laboratory [362].

In a common yeast assay that measures the strength of a compound’s estrogenic response, mixtures of low concentrations (below the ‘no observed effect concentrations’ or NOEC) of chemicals similar to that found in sunscreens demonstrated additive synergistic effects. Other studies indicate that in addition to acting like estrogen in many cellular pathways, compounds found in sunscreens can also antagonize the effects of natural estradiol in other pathways [360].

Application of OMC to the skin of the rats enhanced the penetration of the endocrine-disrupting herbicide 2,4-D [363].

#### Perfluorooctanoic acid (PFOA) and Perfluorooctanoic sulfate (PFOS)

Perfluorooactanoic acid (PFOA) and perfluorooctanoic sulfate (PFOS) are used extensively in commercial applications for their chemical properties of being highly stable and having low surface tension. PFOA is found in compounds such as Teflon® and Gore-tex® as well as in other products including carpet and furniture protectants. PFOS is the main ingredient in Scotchguard® and other products aimed as treatments to provide resistance to soil or stains, especially in textiles.

PFOA and PFOS are ubiquitous, with measurable levels found in wildlife across the planet [364]. These chemicals are found in serum samples from over 95% of the U.S. adults tested in a NHANES study, although levels of PFOS have been decreasing over the past decade as the chemical has been phased out of use in the U.S. [365]. Another study found PFOA and PFOS in blood serum samples taken from adults from nine countries representing four continents [366]. In a study of umbilical cord blood samples from newborns in Baltimore, 100% of the samples had measurable levels of PFOA and PFOS [367]. Higher levels of the chemicals in cord blood were associated with both lower birth weight and smaller size, indicating an effect of PFOA on prenatal development [368]. There are strong correlations between maternal serum and amniotic fluid levels of PFOA. PFOS was also detected in the amniotic fluid, but not until maternal levels were relatively high [369].

Prenatal exposures to PFOA and PFOS have been associated with lower weights at birth, but higher weights at age 20 months in girls participating in the Avon Longitudinal Study of Parents and Children [370]. Follow-up of these same girls when they were 15 years old indicated that these prenatal exposures were associated with increases in serum testosterone levels in teens [371]. Testosterone and other androgens inhibit normal mammary development during adolescence [372].

In southeastern Ohio adolescents exposed to the perfluorinated chemicals, higher levels in blood serum were associated with delayed onset of menstruation in girls [373]. Other studies of Ohio girls demonstrated that exposures to higher levels of PFOA were associated with both later menarche [374] and later breast development [375]. While earlier breast development is a known risk factor for breast cancer, these data support a potential endocrine-disrupting effect of PFOA, which may lead to other health effects later in life. For example, higher levels of serum PFOA (or PFOS) are associated with longer delays in becoming pregnant in women trying to conceive [376].

Higher blood serum levels of PFOA and PFOS, as well as other perfluorinated compounds, were associated with an increased incidence of breast cancer in a study of Inuit women in Greenland. Levels of polychlorinated biphenyls (PCBs) were also elevated in the women who had been diagnosed with breast cancer [377].

In a series of studies examining the effects of gestational or neonatal exposures of mice to PFOA, abnormalities in the formation of mammary tissue were found in the dams during lactation as well as in the pups when they matured. Low-dose exposures to PFOA during pregnancy led to impaired differentiation during lactation, a process that is necessary for normal production and release of milk. In the female pups, mammary glands showed stunted development of epithelial branches before the animals had even been weaned [378]. In a follow-up study that used a cross-fostering design, pups exposed either in utero or in early postnatal life had enduring abnormalities in the development of their mammary tissues, and these abnormalities remained at least through the time of puberty, the latest time evaluated [379]. In a third study, gestational exposures to PFOA were shown to alter mammary development over three generations. In another group of mice, chronic exposures to PFOA in drinking water at levels similar to what is found in some contaminated human water supplies, led to similar negative developmental outcomes in the mammary tissues of the developing pups [378].

The complexity of PFOA’s effects is underscored by a study examining low-dose exposures of different mouse strains to the chemical in the period between weaning and puberty. In one strain (Balb/c), exposures to the chemical led to deficits in normal mammary development through puberty, while in the other strain (C57/BL6), low doses of PFOA exposure enhanced mammary development but higher doses were inhibitory [380]. It is not yet known what factors underlie the strain and dose differences.

On the other hand, when either CD-1 or C57/BL6 mice were exposed to low doses of PFOA prenatally, delays in mammary development were observed in both strains even though there were no observed differences in either ovarian hormone levels or time of onset of puberty. Importantly, these results indicate that the mammary gland is more sensitive to prenatal perturbations by PFOA than are other measures of pubertal status [381].

In hormone-dependent T47D human breast cancer cells, neither PFOA nor PFOS, by themselves, affected cell proliferation. However, in the presence of estradiol, both chemicals did enhance the effects of estradiol on cell growth as well as the expression of several estrogen-responsive genes and ERK1/2 activation [382].

#### Polycyclic aromatic hydrocarbons (PAHs)

Polycyclic aromatic hydrocarbons (PAHs) are ubiquitous byproducts of combustion, which enter the body from sources as varied as coal and coke burners, diesel-fueled engines, grilled meats and cigarettes. PAH residues are often associated with suspended particulate matter in the air, so inhalation is a major means of PAH exposure [383]. In a Silent Spring Institute study of environmental contaminants in house dust, three PAHs (pyrene, benz[a]anthracene and benz[a]pyrene) were found in more than three-quarters of the homes tested [209]. Although they are still found extensively in suspended particulate matter, federally imposed standards on vehicular emissions have led to a significant decrease in PAH release by vehicles compared to their highest levels in the 1970s [384].

Like many other environmental chemicals that are associated with breast cancer risk, PAHs are lipophilic and are stored in the fat tissue of the breast [385]. PAHs have been shown to increase risk for breast cancer through a variety of mechanisms. The most common PAHs are weakly estrogenic [386]. However, the major receptor-directed pathway is through interaction with a protein called the aryl hydrocarbon receptor (AhR), initiating a series of cell changes that lead to altered cell signaling and ultimately to increases in DNA mutations [387, 388]. Although it is currently unclear what the naturally occurring ligand for the AhR is, evidence suggests that the AhR system is important in regulating responses to cellular stress that can lead to disruption of normal cell functioning [389]. At least some of the effects of PAHs are mediated through complex interactions between the AhR-regulated and estrogen-receptor-regulated pathways [390]. PAHs can also be directly genotoxic, interacting directly with the genome and causing damage to DNA [391].

Several epidemiological studies have implicated PAH exposure in increased risk for breast cancer. For example, using traffic exposures estimates of one of the most potent PAHs (benzo[a]pyrene) as a proxy for all traffic related PAHs, researchers compared breast cancer incidence in women exposed to the top 5% of traffic exposure to those with below median exposure levels. Higher exposures to traffic-generated PAHs were associated with an increased incidence of breast cancer in women who ate low levels of fruits and vegetables (OR = 1.46; 95% CI = 0.89–2.40). A significant association was not found for women who ate high levels of these foods. The association with higher incidence of breast cancer was found only for ER−/PR− tumors [392].

In a case-control study, burning synthetic logs, but not wood-only logs, in woodstoves or fireplaces was also associated with an increased risk of breast cancer (OR = 1.42; 95% CI = 1.11–1.84). The association was stronger in women whose exposure was after age 20 and for at least 7 years duration of use. Women who burned synthetic logs and developed breast cancer were more likely to have at least two genetic variants in genes that regulate glutathione-S-transferases, enzymes which are important as cell detoxifiers (OR = 1.71; 95% CI = 1.09–2.69) [393].

One of the studies from the Long Island Breast Cancer Study Project found that women with the highest level of PAH-DNA adducts had a 50% increased risk of breast cancer [394]. More specifically, when PAHs interact with DNA and form an adduct, the result is the loss of one of the purine bases which, when not corrected, leads to gene mutations. These unstable PAH-adducts have been linked to the development of cancer [395].

In an earlier report, researchers explored the presence of PAH-DNA adducts in breast samples taken from women diagnosed with cancer as compared with those diagnosed with benign breast disease. Cancerous samples were twice as likely to have PAH-DNA adducts as were benign samples [396]. Follow-up work indicates that those women who had higher levels of PAH-DNA adducts may not necessarily have had higher exposures to PAHs, but instead were more sensitive to the exposures to PAHs because they had particular genetic profiles that encouraged the deficits in DNA repair [397]. Other studies support the existence of different genetic profiles in women who have increased numbers of PAH-DNA adducts, including polymorphisms in genes involved in cell metabolism, tumor-suppressor mechanisms and DNA repair [397, 398]. Differences were not found in the profiles of genes whose products are involved in the activation and deactivation of the PAHs themselves [399]. A population-based case-control study found that exposures to PAHs were associated with specific mutations of the p53 tumor suppressor gene in breast tumor samples [400].

Occupational exposure studies have looked at workers exposed regularly to gasoline fumes and vehicular exhaust, major sources of PAHs (as well as of benzene). These occupational exposures are associated with an increased risk of breast cancer for pre-menopausal women (low level exposure, OR = 1.56; 95% CI = 0.78–3.12; high dose exposure, OR = 2.40; 95% CI = 0.96–8.01) [401] and also for men. In the case of male breast cancer, PAHs may increase the risk of breast cancer specifically in men carrying a *BRCA1* or *BRCA2* mutation. In a small case-only study of 23 men with breast cancer, four of whom were carriers of a *BRCA1/2* mutation, *BRCA1/2* carrier status interacted with history of ever having driven trucks professionally to enhance the risk of developing breast cancer (COR = 25.5; 95% CI = 1.1–1415) [402].

A case-control study in western New York indicated that very early life exposure (around the time of birth) to high levels of total suspended particulates, a proxy measure for PAH levels, is associated with increased risk of breast cancer in post-menopausal women [375]. An extension of this study, examining PAH exposures at critical times in women’s reproductive histories, demonstrated a relationship between particulate exposures around the time of the first menstrual period and incidence of pre-menopausal breast cancer (OR = 2.05; 95% CI = 0.92–4.54), and a relationship between exposure level at the time a woman first gives birth and her risk of post-menopausal breast cancer (OR = 2.57; 95% CI = 1.16–5.69) [403].

The studies above all looked at breast cancer incidence. Two reports examined the relationship between PAH exposures and mortality. Using an ecological model exploring the association between suspended fine particulate matters in several municipalities in Taiwan, researchers found that women living in areas with high levels of particulate matter in the air had an increased probability of dying from breast cancer, as compared to those living in cleaner areas (RR = 1.19; 95% CI = 1.03–1.38) [404]. Another report examined PAH-DNA adduct levels and mortality among women who had been diagnosed with breast cancer. In an extension of the Long Island study described above, researchers found no overall relationship between survivorship and PAH-DNA adduct levels. Looking more closely at groups of women who had undergone different types of treatments, however, revealed a twofold increase in age-adjusted mortality rates from breast cancer among women with high PAH-DNA adduct levels who had received radiation treatment (HR = 2.47; 95% CI = 0.74–8.21). However, there was an increased survival rate for women with adducts who had received hormone therapy as part of the treatment for their breast cancers (HR = 0.52; 95% CI = 0.24–1.13) [405].

#### Pesticides and herbicides

A 2007 report from the Long Island Breast Cancer Study Project demonstrated that self-reported lifetime use of residential pesticides was associated with an increase in risk for breast cancer. The increase was found for women who had reported use of pesticides in the aggregate (ever having used any residential pesticides; OR = 1.39; 95% CI = 1.15–1.68), as well as specifically for use of lawn (OR = 1.48; 95% CI =1.20–1.82) and garden (OR = 1.58; 95% CI = 1.12–2.22) pesticides, although there were no relationships between perceived doses of exposures and risk for cancer [406]. These results are important because they address exposures to chemicals in the course of ordinary life, with all the complexities of mixtures and multiple sorts of uses. Many other studies focus on single chemicals or classes of chemicals, and the results are often contradictory depending on length and timing of exposures, types of chemical being studied and so forth. Despite that, many pesticides and herbicides have been labeled as human or animal carcinogens. Many are found in water supplies [406] as well as samples of air and dust from homes [209, 407].

##### Triazine herbicides: atrazine

Triazine herbicides (including atrazine, simazine, and cyanazine) are the most heavily used agricultural chemicals in the United States. Atrazine and simazine have been banned in the European Union, and cyanazine is labeled as a highly toxic mutagen, because of their high presence in drinking water, demonstrated harmful effects on wildlife, and potential health effects in humans. Cyanazine was phased out of use in the U.S beginning in 1996, although simazine and atrazine are still approved for use in the United States. More than 75 million pounds of atrazine, the most heavily used of the chemicals, were applied annually in the United States in 2008 (the most recent year for which the Environmental Protection Agency (EPA) has data), primarily to control broadleaf weeds in corn and sorghum crops in the Midwest [408].

Elevated levels of atrazine are found each spring and summer in both drinking water and groundwater in agricultural areas [409–411]. Atrazine is a known endocrine disruptor, causing dramatic damage to reproductive structures in frogs, fish and other wildlife [412, 413]. Although all three triazines have been shown to cause mammary cancer in laboratory rats [414] and increased proliferation of human breast cell lines in vitro [415], there is relatively little scientific data exploring the relationship between simazine or cyanazine and human breast cancer. The literature on atrazine is somewhat more substantial.

High levels of triazines, mainly atrazine, in contaminated waters were associated with an increased risk (OR = 1.20; 95% CI = 1.13–1.28) of breast cancer [416] although a further expansion of this study concluded that there was no relationship between atrazine exposures and risk for developing breast cancer [417]. Similarly contradictory results were found in another ecological study in which higher levels of mixed pesticides, including atrazine, were associated with increased breast cancer in one rural county in the UK, but not in the neighboring county [418]. Because these ecological studies tend to compare countywide average levels of atrazine contamination and incidence rates, rather than individual exposure histories and health outcomes, it is difficult to understand clearly the difference in results [419].

A weight-of-the evidence review of seven epidemiological studies, funded by Syngenta — the producer of atrazine, concluded that across study designs, there was no causal relationship between exposures to atrazine and development of breast cancer. However, availability of only relatively few studies, lack of attention to breast cancer subtypes and other methodological complications makes ruling out an association unsupportable [420].

Research in rodents has shown that atrazine exposure disrupts pituitary-ovarian function, resulting in decreases in circulating prolactin and luteinizing hormone levels, changes that contribute to the effects of this herbicide on increases in mammary tumors [414, 421]. Atrazine also exerts endocrine-disrupting effects by increasing the activity of the enzyme aromatase [422, 423], an enzyme that catalyzes the conversion of testosterone and other androgens to estrogens, including estradiol.

Exposure to atrazine or mixtures of atrazine metabolites during gestation delays development of the rat mammary gland in puberty, widening the window of sensitivity to breast carcinogens [424–426]. Similarly, exposure of rats late in pregnancy to a mixture of commonly formed metabolites of atrazine also leads to persistent changes in mammary gland development in pups exposed during gestation. These abnormalities persist into adulthood. Exposure of rats with existing mammary tumors to atrazine increases the rate of cell proliferation in those tumors [427]. The early changes in mammary gland development may reflect an indirect effect of atrazine on maternal health, especially atrazine-induced caloric restriction, during the time of pesticide exposure [428].

Although atrazine is an endocrine disruptor in estrogen-directed pathways, several studies have indicated that atrazine does not exert its effects through binding to ER [429, 430]. Other proposed mechanisms by which atrazine may alter estrogen pathways include through binding to and increasing expression of the membrane-bound GPR30 receptor [407, 423], activation of the steroidogenic factor-1 (SF-1) gene, activation of ERK phosphorylation, and direct or indirect amplification of cAMP [430–432].

##### Heptachlor

Heptachlor is an insecticide that was widely used in the United States throughout the 1980s, especially for termite control. In 1988, the U.S. EPA restricted use of heptachlor to certain applications for controlling fire ants, but agricultural use continued until 1993 because growers were allowed to use up existing stocks [433]. Heptachlor use was particularly high in Hawaii, where it was employed extensively on pineapple crops and consequently contaminated both local agricultural crops and dairy supplies. Breast cancer rates in Hawaii have increased dramatically for women of all ethnic groups over the past four decades [434]. In a relatively small (96 cases) case control study exploring possible relationships between serum levels of organochlorine pesticides, including heptachlor, and development of breast cancer, a trend (*P* = .078) between heptachlor concentrations and breast cancer risk was found.

Heptachlor still contaminates both soil and humans. Its breakdown product, heptachlor epoxide (HE), is known to accumulate in fat, including breast tissue. Levels are highest in women ages 20 and older, but HE is also found in the bodies of adolescents 12 to 19 years old [435] and in eight of ten samples of umbilical blood from newborn infants [436]. High levels of heptachlor in breast milk [437] and fat tissue from breast biopsies [438] have been shown to be associated with increased incidence of breast cancer.

Although HE does not act like estrogen, it affects the way the liver processes hormones, thereby allowing levels of circulating estrogens to rise and increasing breast cancer risk. HE also has been shown to disrupt cell-to-cell communication in human breast cells in culture [439] and to increase production of nitric oxide, a chemical that is found naturally in cells and known to cause damage to DNA [438].

##### Dieldrin and aldrin

From the 1950s until 1970, the pesticides dieldrin and aldrin (which breaks down to dieldrin, the active ingredient) were widely used for crops including corn and cotton. Because of concerns about damage to the environment and, potentially, to human health, in 1975 the EPA banned all uses of aldrin and dieldrin except in termite control; the EPA banned these pesticides altogether in 1987 [440]. Thus, most of the human body burden of this chemical comes either from past exposures or lingering environmental contamination.

Hoyer et al. showed a clear relationship between breast cancer incidence and dieldrin in their examination of a rare bank of blood samples taken from women before the development of breast cancer [441]. During the late 1970s and early 1980s, blood samples were taken from approximately 7500 Danish women age 30 to 75. Researchers detected organochlorine compounds in most of the 240 women who were diagnosed with breast cancer prior to the study’s publication in 2000. They found dieldrin in 78% of the women who were later diagnosed with breast cancer, with women who had the highest levels of dieldrin before diagnosis having more than double the chance of developing the disease than women with the lowest levels. Exposure to dieldrin correlated with the aggressiveness of breast cancer: highest levels of dieldrin were associated with higher breast cancer mortality (RR = 2.61; 95% CI + 0.97–7.01; P_trend_ = <.01) [442].

Treatment of mice prenatally and neonatally to environmentally relevant doses of dieldrin increased the number and size of mammary tumors. These effects may have been mediated through changes in the cellular expression of the growth factor BDNF and cell-signal receptor Trks. Both of these were elevated in tumors from the dieldrin-treated animals [443].

Like many other pesticides found in the environment, dieldrin has been shown to be an endocrine disruptor, both by stimulating estrogen-regulated systems and by interfering with androgen-regulated pathways. Addition of dieldrin to human breast cancer (MCF-7) cells in vitro can stimulate their growth and proliferation [444].

##### Other pesticides

A case-control study of 128 Latina agricultural workers newly diagnosed with breast cancer in California and 640 cancer-free controls, identified three pesticides—chlordane, malathion and 2,4-D—associated with an increased risk of the disease. Scientists found that the risks associated with use of these chemicals were higher in young women and in those with early-onset breast cancer than in unexposed women [445].

Engel et al. studied the association between pesticide use and breast cancer risk in farmers’ wives in the NCI’s Agricultural Health Study. This large prospective cohort study enrolled more than 30,000 women in Iowa and North Carolina. Researchers found evidence of increased incidence of breast cancer in women using 2,4,5-trichlorophenoxypropionic acid (2,4,5-TP) (RR = 2.0; 95% CI = 1.2–3.2); a non-significant association was found for dieldrin and captan. Incidence was also modestly elevated in women whose homes were closest to areas of pesticide application (RR = 1.7; 95% CI = 1.0–1.9) [446].

Young children of farmers using 2,4,5-TP on their farms had high levels of the pesticide in their urine samples soon after the chemical had been applied to the fields [447]. This is of concern given the evidence of increased susceptibility of children and young adolescents to the carcinogenic effects of endocrine disrupting chemicals.

Treatment of female rats with malathion resulted in abnormal increases in mammary duct proliferation and induction of mammary tumors when animals were tested at 8 months of age [448].

#### Persistent organochlorines

##### DDT/DDE

Dichloro-diphenyl-trichloroethane (DDT) was the first widely used synthetic pesticide. It is credited on the one hand with the eradication of malaria in the United States and Europe and on the other with long-term devastating effects on reproductive success in wildlife and adverse health effects in humans [449]. Banned in most countries for agricultural use, DDT is still used for malaria control in many countries, especially in sub-Saharan Africa [450]. Because of its continued use and its persistence in the environment, DDT, and its main metabolite, DDE, are found worldwide. Most animals, including humans, ingest DDT and DDE-contaminated foods and retain the chemicals. Significant concentrations of DDT and DDE are found in the body fat of humans and animals as well as in human breast milk and placenta, even in regions where it has not been used for a long time [452–454].

Epidemiological data are mixed regarding the effects of DDT/DDE on breast cancer risk [454]. A case-control study from Tunisia found positive associations between serum DDE levels and breast cancer risk (OR = 9.65; 95% CI = 1.81–63.33; dose-response trend *p* = .02). On the other hand, a study from the Long Island Breast Cancer Study Project did not find an association between DDT/DDE (or polychlorinated biphenyls, PCBs) and breast cancer [456]. Both of these studies measured contaminant levels around the time of breast cancer diagnosis, without regard to possible exposures during critical early periods of breast development [457].

Two critical studies that examined early life (prenatal and childhood) exposures to DDT have demonstrated a clear association between exposures to DDT and increased risk for developing breast cancer [103, 104]. A prospective, nested case-control study of 129 women who had been diagnosed with breast cancer before age 50 and 129 age-matched controls, all participating in the Child Health and Development Studies (CHDS), explored the women’s estimated historical DDT levels based on aggregate data from their year of birth as well as blood DDT levels at the time the women gave birth to their first child. Researchers then monitored the women for the next 2 decades, noting when women either were diagnosed with breast cancer (invasive or noninvasive) before age 50 or died from breast cancer before age 50. Exposure to DDT during childhood and early adolescence (younger than 14 years) was associated with a 5-fold increase in the risk of developing breast cancer before age 50 [104].

In a case-control prospective study of 9300 women in the CHDS pregnancy cohort (daughters of the mothers in the larger CHDS cohort), stored postpartum maternal blood samples were analyzed for levels of DDT. Daughters were followed for 52 years and breast cancer diagnosis in this cohort was determined. DDT levels in perinatal blood of mothers from breast cancer cases were compared with levels in perinatal blood samples from mothers of age-matched controls. Higher maternal DDT levels were associated with a significant increase in occurrence of breast cancer in their daughters by age 52 (OR = 3.7; 95% CI = 1.5–9.0) [103].

A comparison of the association between disease risk and DDT use in developed countries (where DDT has been banned for several decades) and in developing countries (where DDT use is still prevalent) supports the premise that exposures to DDT are associated with increased risk of breast cancer. The association between DDT levels and breast cancer was much stronger in developing countries, where women of the age to be diagnosed with breast cancer also would have been exposed to DDT during critical periods of development [458].

A study from the Long Island Breast Cancer Study Project examined post-diagnosis mortality and serum DDT levels at time of diagnosis. Higher levels of DDT 5 years after diagnosis were associated with an increased mortality rate (HR = 2.72; 95% CI = 1.04–2.12) although the effect was not significant at 15 years post diagnosis [459].

Laboratory studies have found that in addition to being directly genotoxic or carcinogenic [460], the estrogen-like form of DDT enhances the growth of ER+ mammary tumors [461–463]. The percentage of breast tumors in the United States that are ER+ rose from 73% in 1973 to 78% in 1992 [464]. Although no direct relationship can be inferred, this change corresponds to the period when women exposed to DDT as young girls were expected to be exhibiting environmentally altered incidence in breast cancer related to DDT exposure. Another study, looking at chemical levels in breast fat tissue, did not find an association of DDT/DDE with ER+ tumors. However, data from this study indicated a significant association of higher concentrations of these compounds in breast tissue with tumors that were more aggressive and that had poorer prognoses (OR = 2.40; 95% CI = 1.0–5.4) [465].

##### PCBs

Although the EPA banned the use of PCBs in new products in 1976, substantial amounts of the insulation fluids, plastics, adhesives, paper, inks, paints, dyes and other products containing PCBs manufactured before the ban remained in use for decades [466]. About one-third was discarded, which means that these toxic compounds eventually made their way into landfills and waste dumps [467]. PCBs are found in the air and in aquifers and rivers, where they accumulate in the sediment but then are re-dissolved into the water where they contaminate and bioaccumulate across the food chain [468, 469].

Levels of PCBs were high before being banned in the United States, but generally their presence in the environment and in human tissues has decreased slowly over the past decades [470, 471]. Choi et al. found that PCB levels in neonatal cord serum were correlated with the distance of mothers’ residences from a Superfund site; levels were lower after site remediation [472]. However, exposure levels were high between childhood and young adulthood for many women who are now facing breast cancer diagnoses.

The more than 200 individual PCBs are classified in three types based on their cellular effects. Group I PCBs are estrogenic; Group II compounds are anti-estrogenic; and Group III PCBs appear not to be hormonally active, but can stimulate the enzyme systems of animals, including humans, in a manner similar to certain drugs (such as phenobarbital) and other toxic chemicals [473, 474]. Additionally, hydroxylated metabolites of PCBs alter the expression of genes involved in hormone synthesis, indicating that these compounds may act as endocrine disruptors through mechanisms not directly involving the estrogen receptor [475].

There are several epidemiological studies that have implicated exposures to PCBs as a risk factor for later development of breast cancer. Women who regularly ate PCB-contaminated pike or perch had higher risk for breast cancer than women who never ate these fish (perch: OR = 7.90; 95% CI = 1.01–61.9; pike: OR =9 .07; 95% CI = 1.10–74.4) [476]. Another study implicated PCBs in breast cancer recurrence among women with non-metastatic breast cancer. The study found that women with the highest levels of total PCBs (RR = 2.91; 95% CI = 1.0–8.2), as well as of PCB 118 (RR = 4.0; 95% CI = 1.3–4.9), in their fat tissues were almost three times as likely to have recurrent breast cancer as women with lower levels [477].

Most studies have looked at total PCB levels without identifying individual types. A few studies, however, have looked at relationships between cancer status and particular PCBs or groups of PCBs. For example, a recent meta-analysis demonstrated that there was no relationship between exposures to Group I PCBs, but there was a significantly increased risk of breast cancer with exposures to either Group II (OR = 1.23; 95% CI = 1.08–1.40) or Group III (OR = 1.25; 95% CI = 1.09–1.43) PCBs [474]. Another study examined PCB levels in breast tissue from disease-free women and women with metastatic breast cancer. Across all samples tested, higher levels of Group III PCBs were found, followed by Group II and then Group I compounds. These results were independent of disease status and were not associated with any pathological characteristics in the women who had been diagnosed with breast cancer [478]. A recent congener-specific meta-analysis examined association between representative congeners from three subgroups of PCBs and found Group III congeners PCB 99 and PCB 183 conferred a greater risk than Group I PCB 187 (respective ORs: 1.36; 95% CI = 1.02–1.80; 1.56; 95% CI = 1.25–1.95; 1.18; 95% CI = 1.01–1.39) [479].

In a case-control study, Aronson et al. measured several types of PCBs, along with DDE, in tissue samples from women scheduled for excision biopsy of the breast. Increased risk for breast cancer was associated with higher concentrations of Group II PCBs 105 and 118, with the ORs for these two PCBs increasing linearly with higher concentrations (*p* for trend <0.01) [480].

On the other hand, some studies have found no link between PCBs and breast cancer [481]. A 2009 review of the literature concluded that the overall picture was that PCBs, as a class (not considering the types of PCBs), were not associated with increased risk for breast cancer [482]. In a study examining occupational exposures to PCBs in electrical capacitor production workers and later breast cancer incidence, no overall relationship between exposure levels or duration and disease incidence was observed for female workers in general. But for non-white women, a significant relationship was found between incidence of breast cancer and earlier PCB exposure duration as well as cumulative exposure amounts (comparing highest vs. lowest categories of exposure, HR = 22.3; 95% CI = 2.38–209) [483]. More recently, Artacho-Cordón found no correlation between serum or adipose levels of PCBs and risk for being diagnosed with breast cancer [484].

Some of these compounds may have their greatest impact on women with particular susceptibilities and looking broadly at large samples will not tell the full story of cancer risk as influenced by PCB exposures. For example, researchers evaluating data from the Nurses’ Health Study revisited the issue of PCBs and breast cancer risk and revised their conclusion concerning the link between PCBs, DDE and breast cancer. In studies of PCBs and DDE in blood, they had previously concluded that exposure to these chemicals was unlikely to explain high breast cancer rates [485]. Newer evidence found that the complex interaction of high serum levels of PCBs and a particular variant (*exon 7*) of the *CYP1A1* gene was associated with an increased risk for breast cancer (HR = 2.78; 95% CI = 0.99–7.82, compared to women with lower PCB levels and the wild-type genotype) [486].

As was true for the critiques of the DDT studies cited above, the methods used to test these relationships do not account for exposures to PCBs during earlier developmental times when mammary tissue is particularly sensitive to the toxic effects of many environmental chemicals [487]. The results from Cohn’s work on DDT and breast cancer make clear that this is a critical methodological issue [457].

##### Dioxins

Dioxins are formed by the incineration of products containing PVC, PCBs and other chlorinated compounds as well as from industrial processes that use chlorine and from the combustion of diesel and gasoline [488]. One of the dioxins (2,3,7,8-tetra chlorodibenzo-para-dioxin; TCDD) has been classified by IARC [489] and the U.S. EPA [490] as a carcinogen.

Dioxins accumulate in the body fat of wildlife and humans, and they break down very slowly, with a half-life of 7–11 years in body tissues [491]. People are exposed to dioxins primarily through consumption of animal products and human breast milk [488, 489]. Dioxin enters the food chain when vehicle exhaust or soot from incinerated chlorinated compounds falls on field crops later eaten by farm animals. It is then passed to humans through dairy and meat products. The body fat of every human being, including every newborn, is thought to contain dioxins [492].

There is a substantial decrease in the amount of dioxin remaining in a women’s breast fat tissue after she has breastfed because the chemicals have been passed on to her newborn via breast milk [493]. Although the presence of toxic chemicals in breast milk is potentially dangerous, the beneficial nutrients and immune system boosters that are transferred from mother to infant are thought to far outweigh the potential toxic transfers [494]. But in addition to potential transfer of dioxins to breast-feeding infants, the release of the chemicals from storage in breast fat cells, initiated by the process of milk synthesis, may actually trigger genotoxic effects in the mother’s breast tissue. Addition of breast milk extracts to MCF-7 cells led to a reprogramming of gene expression to a pattern typically found following estrogen stimulation, especially in the *CYP1A1* and *CPY1B1* genes [495].

A study of women exposed to dioxins during a chemical plant explosion in 1976 in Seveso, Italy demonstrated a time-dependent association between dioxin exposure and breast cancer [496, 497]. A tenfold increase in TCDD levels in blood samples taken at the time of the explosion was associated with more than twice the risk of breast cancer in the women who, in 1996, averaged 40 years old (HR = 2.1; 95% CI = 1.0–4.6), and whose breast cancer was diagnosed pre-menopausally. Follow-up of the cohort in 2008 revealed a non-significant increase in risk for developing breast cancer between the time the women were 40 and 12 years later, when they were 52, on average (HR = 1.44; 95% CI = 0.89–2.33). For all the breast cancer cases for which there were cancer profile data, more than 80% were ER+/PR+ cancers [497]. Continued follow-up of this cohort will examine risk of developing breast cancer as the women enter and continue into the post-menopausal years.

A retrospective mortality study in Germany examined deaths from cancer among people who had worked in a chemical factory in which they were exposed to high levels of TCDD. As compared with national mortality rates in West Germany, 5 years after closure of the plant, there was no increase in overall mortality from cancer for female workers, although there was a significant increase in deaths from breast cancer among those who worked in high-exposure regions of the factory (SMR = 2.15) [498]. A later follow-up 23 years after the closing of the plant found a lower rate of mortality from all causes for women workers in the plant (SMR = 0.91; 95% CI =0.78–1.05), but a significant increase in mortality from breast cancer (SMR = 1.86; 95% CI = 1.12–2.91) in this cohort [499].

Several laboratory studies have demonstrated that the timing of exposures to dioxins matters. Although exposing animals to dioxins in adulthood may not affect cancer rates, earlier exposures may have profound effects. Administration of dioxins (especially TCDD) to pregnant rats leads to structural abnormalities in the development of their pups’ mammary tissues and higher incidence of tumors when the pups grow to adulthood [500–504]. TCDD may exert its cancer-causing effects both by decreasing the efficacy of tumor-suppressor mechanisms and by enhancing the estrogenic signaling within the mammary cells [505].

TCDD has been shown to inhibit estradiol-induced cell growth and proliferation as well as other pathways regulated by estrogens, including methylation of CYP1A1, in a variety of human breast cancer cell culture lines [506, 507]. Like the PAHs described above, dioxins like TCDD exert their effects by activating both the aryl hydrocarbon receptor (AhR), an important mediator of cell growth and proliferation [508] and anti-apoptosis pathways [509], as well other AhR-independent pathways including PR- mediated pathways [506, 510, 511]. Increasing evidence indicates that many of the effects of TCDD and other dioxins is the result of cross-talk between the AhR and ER, PR and even AR systems [512, 513].

Prenatal treatment of rats with low doses of TCDD led to increases in the number of terminal end buds (TEBs) counted in whole mount preparations of postnatal day 71 females. The treatment also resulted in a suppression of *BRCA-1* expression, a result of increased *BRCA-1* promoter hypermethylation [514].

#### Polybrominated Diphenyl Ether (PBDE) fire retardants

PBDEs are a complex group of chemicals that are structurally similar to the polychlorinated biphenyls (PCBs). Products containing PBDEs include polyurethane foam in furniture (penta-BDE) and electronic and plastic products (octa- and deca-BDEs) [515].

Although both penta- and octa-BDEs have been banned in the European Union and have not been produced in the United States since 2004, products containing them remain throughout the world. PBDEs are found ubiquitously in the environment and are detected in air, dust, soil and food as well as in many wildlife species. Although home exposures (as measured by dust levels) have decreased over the past decade, levels remain high enough to remain a serious health concern [516].

There is considerable geographic variability in exposures to the chemicals; people in California, with its historically stringent furniture flammability standards, have much higher levels of PBDE exposures than do people in Massachusetts. Within the California group, lower socioeconomic status was associated with higher PBDE levels [488, 517]. Mexican Americans living in California have significantly higher PBDE levels in blood serum than do Mexicans living in their homeland [518].

Data from young girls (ages 6 to 9) from California and Ohio support these findings. Although PBDEs were found in almost all samples tested, girls in California had significantly higher blood serum PBDE levels than did girls from Ohio, and young black African American girls had higher levels than either white or Hispanic girls [519]. In these cohorts, PBDE exposures are associated with delays in puberty in adolescent girls (RR = 1.05; 95% CI = 1.02–1.08), an effect that is not moderated by adjustment for BMI [520].

PBDEs have been found in human fat tissue, as well as in blood serum, breast tissue and milk [521–523]. PBDEs cross the placenta, resulting in exposures to developing fetuses [524]. PBDEs are endocrine-disrupting compounds, exerting effects on a number of hormonal systems, including the androgens, progestins and estrogens, although the major system affected by PBDEs is the thyroid hormone system [486]. Most studies of health outcomes after PBDE exposures have focused on neural development, given the prominent role of thyroid hormones (especially T_4_) in regulating brain development [525, 526].

Very few data directly address the possible effects of PBDEs on breast cancer risk. One case-control study found no relationship between PBDE levels in breast fat and breast cancer risk, but the sample was small and the chemical analysis was done around the time of diagnosis of breast cancer in the women who developed the disease [527]. However, at least some PBDEs have been shown to be as effective as many of the other endocrine-disrupting compounds in promoting estrogenic-like proliferation of human breast cancer cells in vitro [528]. Penta-BDE enhances tumor-cell proliferation in MCF-7 cells through estrogen-like effects on cell pathways that interrupt apoptosis [529]. The cell-proliferative and anti-apoptotic effects of PBDEs are additive with those of natural estradiol [530] and counteract the anti-cancer effects of tamoxifen in cultured breast cancer cells [531]. Given the extensive overlap and interaction of estrogen-mediated and thyroid-mediated responses in the regulation of breast cancer [532], PBDEs will be a class of chemicals of continued concern for scientists interested in understanding environmental links to breast cancer [533].

Even as PBDEs are being used less often as fire retardants in common consumer products, there is now evidence that the chemicals being used as substitutes — including Firemaster 550®, a common substitute with proprietary ingredients — are increasingly contaminating our environment [489, 534]. Although the physiological effects of exposures to Firemaster 550® have not yet been studied extensively, one study demonstrated that feeding rat dams low doses during pregnancy and lactation led to changes associated with exposures to other endocrine disrupting compounds. These effects included changes in thyroid hormone levels in the mothers, and changes in behavior, weight gain and earlier puberty in female pups [507].

#### Aromatic amines

Aromatic amines are a class of chemicals found in the plastic and chemical industries as byproducts of the manufacturing of compounds such as polyurethane foams, dyes, pesticides, pharmaceuticals and semiconductors [535]. They are also found in environmental pollution such as diesel exhaust, combustion of wood chips and rubber, tobacco smoke and grilled meats and fish [536, 537]. There are three types of aromatic amines: monocylic, polycyclic and heterocyclic.

Three monocyclic amines, including o-toluidine, have been identified in the breast milk of healthy lactating women [536], as well as in the urine of most people [535]. σ-Toluidine is known to cause mammary tumors in rodents [538, 539]. The carcinogenic aromatic amines, 2-amino-phenylimidazo[4,5-b]pyridine (PhIP) and 4-aminobiphenyl (ABP) are also found in human breast milk, as are DNA-adducts of these compounds [540].

Occupational exposures of female rubber-factory workers to another set of monocyclic aromatic amines derived from p-phenylenediamine are associated with an increased risk of breast cancer in the following several years. The amount of increased risk was correlated with total cumulative exposure levels to the aromatic amines, with lowest levels leading to a 3.7-fold increase in cancer and the highest levels of exposure increasing risk more than tenfold [541].

A case control study of women who used hair dyes, in comparison with those who have not, revealed an increased risk of breast cancer in the dye users (OR = 1.15; 95% CI = 1.06–1.24). In addition to intentional inclusion of p-phenylenediamine [542], major contaminants in many hair dyes include PhIP and ABP [543].

Heterocyclic aromatic amines (HAAs) are formed (along with PAHs) when meats or fish are grilled or otherwise cooked at high temperatures. In a case control study, Steck et al. found an association between higher lifetime consumption of grilled meats and fish and increased incidence of post-menopausal breast cancer (OR = 1.42; 95% CI = 1.12–1.92) [544]. Studies of both milk and cells from the ducts of women’s breast revealed the presence of DNA adducts in association with HAAs [545, 546].

Aromatic amines are metabolized by N-acetyltransferases. This metabolic process ultimately leads to other compounds that are thought to be the actual carcinogenic chemicals. There is an extensive literature examining whether or not genetic profiles that alter the efficacy or speed of N-acetyltransferase activation, especially through the N-acetyltransferase 2 (NAT2)-regulated pathway, might alter susceptibility to breast cancer. Studies have reached differing conclusions about the role of possible polymorphisms of the NAT2 gene on breast cancer susceptibility. A 2010 study tried to disentangle many of the possible confounding factors and found that eating grilled meat (and drinking coffee) resulted in greater risk for diagnosis of ER- breast tumors in women with the ‘slow-acetylator’ form of the NAT2 gene [547].

Laboratory studies of HAAs in systems using cultured breast cancer cells demonstrated that these chemicals can mimic estrogen, and they also can have direct effects on cell division processes in ways that might enhance the development of tumors [548].

#### Metals

Higher accumulations of iron, nickel, chromium, zinc, cadmium, mercury and lead have been found in cancerous breast biopsies as opposed to biopsies taken from the breasts of women without breast cancer. These metals also have been found in higher concentrations in serum and urine from women diagnosed with cancer as compared with those from healthy women [549–552].

Laboratory studies have shown that a number of metals including copper, cobalt, nickel, lead, mercury, methylmercury, tin, cadmium and chromium have estrogenic effects on breast cancer cells (MCF-7) cultured in vitro [553–555], with cadmium expressing the highest level of estrogenic activity [555]. The most extensive work on the relationship between breast cancer and metals has been done examining this metaloestrogen, cadmium.

Several epidemiological studies have demonstrated an association between higher cadmium levels in urine (OR = 2.29; 95% CI = 1.3–4.2) [556, 557] or blood [558] and increased breast cancer risk, although in a large prospective study using the WHI cohort, no association between urinary cadmium levels and risk for developing breast cancer was found [559]. Nevertheless, a recent meta-analysis reported a significant increase in risk of developing breast cancer as urinary cadmium levels increased (OR = 2.24; 95% CI = 1.49–3.35) [560].

Differences in the efficacy of establishing relationships between breast cancer and cadmium exposures as determined by dietary vs. urinary cadmium measures may reflect the observation that urinary cadmium levels are a stronger marker of lifetime exposures to the metal, given cadmium’s half-life of 12–30 years, while dietary exposure levels reflect a shorter term and potentially more variable marker [560].

Prospective studies of women’s dietary intake of cadmium and later diagnosis of cancers demonstrated a significant relationship between higher levels of dietary cadmium exposure and incidence of both endometrial cancers (RR = 1.39; 95% CI = 1.04–1.86) [561] and post-menopausal breast cancers (RR = 1.21; 95% CI = 1.07–1.36) [562]. With regard to breast cancer, the effect was significant for all sub-types combined, but more pronounced for ER+ tumors (OR = 1.94; 95% CI = 1.04–3.63 [563]; RR = 1.23; 95% CI = 1.02–1.49) [562]. On the other hand, studies examining dietary cadmium intake in Japanese [563, 564] and Danish [565] women and their risk of developing breast cancer found no relationship. A 2012 study in the United States that looked at dietary cadmium levels and breast cancer risk also did not find a significant relationship [566], nor did two recent meta-analyses that studied this relationship [567, 568].

In young rats, treatment with low doses of cadmium led to an increase in branching and bud formation in mammary tissue, and the induction of several estrogen-associated proteins. Prenatal exposure of rats to cadmium led to early onset of puberty and greater numbers of mammary terminal end buds, both known risk factors for breast cancer [569].

Estrogenic effects of cadmium have been studied in some detail, and the metal has been shown to interfere with a number of normal estrogen-sensitive pathways [570]: cadmium can bind to and activate mammary cell estrogen receptors; it also interacts with and regulates the transcription of estrogen-dependent genes affecting the synthesis of proteins and/or the activity of cell-signaling pathways in ways similar to the natural estrogen, estradiol [570, 571]. Cadmium exposure can also lead to malignant transformation of normal human breast epithelial cells (MCF-10A) through a mechanism that does not require the presence of ERα [572]. Other studies support the possibility that cadmium may also exert cellular effects through mechanisms beyond the conventional nuclear-ER directed pathways [573, 574], including possibly through binding to the membrane receptor, GPR30 [575].

In addition to hormone-mediated effects, cadmium may also promote the development of cancer through epigenetic processes including changes in DNA methylation patterns as well as possible modifications of gene-associated histones [576].

Like several other estrogen-mimicking endocrine disruptors, cadmium interferes with the efficacy of a common chemotherapeutic agent often prescribed for women who have been diagnosed with breast cancer. In a study examining the effects of cadmium, 5-fluorouracil (5-FU), and the two combined on subcellular structure and metabolic activity in cultured MCF-7 breast cancer cells, co-administration of cadmium negated the anti-cancer effects of 5-FU [577].

*Section summary:* The growing literature on exposures to EDCs, especially early in development, indicates an increased risk of developing breast cancer following exposure to many of these compounds, either alone or in combination. The most substantial human epidemiological data supporting this relationship come from prospective studies on women exposed to DDT during gestation or early childhood and increased development of premenopausal breast cancer [103, 104]. The largest non-human literature connects early, low-dose exposures to BPA to increased risk for developing mammary tumors. Both in situ and in vitro studies have added substantially to our understanding of the complex mechanisms underlying these relationships. Although not as robust as for the above chemicals, links between exposures, especially early in development, and many other EDCs have also been documented.

### Hormones in foods: natural and additives

The prevailing evidence against synthetic estrogens must also be understood alongside evidence about the effects of plant estrogens (phytoestrogens) on risk for developing breast cancer. While most of the research in this area has focused on possible protective associations with soy-based isoflavones in a normal dietary regime, a growing literature is also examining the potential protective effects of the lignans, enterolactone and α-linolenic acid.

Mycoestrogens (estrogens found in fungal species) can contaminate agricultural and meat products, and this contamination may increase susceptibility to developing breast cancer. Also, exposures to growth enhancing compounds given to meat-producing and dairy animals have been linked to increased risk for developing breast cancer. This section explores the complicated profiles of exposures to food-based estrogens and risk for developing breast cancer. There has been no dermination on potential carcinogenicity of these substances by IARC or NTP.

#### Phytoestrogens (plant estrogens)

Foods such as whole grains, dried beans, peas, fruits, broccoli, cauliflower and especially soy products are rich in phytoestrogens. While most of the research in this area has focused on possible associations with soy-based isoflavones. Additionally, there is also a growing literature is also examining the possible role of the lignans, including enterolactone and α-linolenic acid, in affecting breast cancer risk.

##### Lignans

Lignans are polyphenolic compounds found widely in seeds and grains common to a Western diet. They are rapidly metabolized in the gut to the estrogenic compounds, enterolactone and enterodiol [578].

A matched case-control study of mainly premenopausal women in the Nurses’ Health Study II cohort found no overall relationship between plasma enterolactone levels and breast cancer risk. However, in those women whose follicular circulating estradiol levels were below the median, higher enterolactone was associate with a significant decrease in breast cancer risk (OR = 0.49; 95% CI = 0.27–0.91) [579]. A meta-analysis examining the possible relationship between serum enterolactone levels and either all-cause mortality or breast-cancer associated mortality found negative correlations between the circulating lignan levels and both outcome measures (HR = 0.57; 95% CI = 0.42–0.67 for all-cause mortality and HR = 0.54; 95% CI = 0.39–0.75 for breast cancer mortality [580].

A recent examination of possible mechanisms underlying the protective effect of enterolactone used the aggressive, highly invasive MDA-MB-321 human breast cancer cell line. Addition of the lignan to the cell culture led to decreased activity in mRNA levels of several genes associated with cell proliferation, as well as increased retention of mitotic cells in the S-phase, and decreased migration and invasion through interference with the cell cytoskeleton [580].

A systematic review of the literature on associations between α-linolenic acid and risk of breast cancer found significant negative relationships between higher intake of flax, a major source of α-linolenic acid, and both breast cancer incidence (OR = 0.82; 95% CI = 0.69–0.97) and mortality (HR = 0.69; 95% CI = 0.50–0.95) [581]. In women with recent diagnoses of breast cancer, higher intake of flax was associated with less-aggressive tumor profiles that had higher apoptotic indices, and lower HER2 expression and proliferative rates [581].

##### Soy and soy derivatives

Although scientific evidence suggests that soy-derived foods offer nutritional benefits and are associated with healthy diets [582, 583], the data are more conflicting as to whether the soy-based diets are beneficial, harmful or neutral when it comes to affecting breast cancer risk [584, 585].

Some of the disparity may be related to type of soy products consumed by individuals. Across Asian and Western diets, soy may be processed in scores of different ways, resulting in up to 100-fold differences in levels of particular phytoestrogens between products [583] and very different exposures levels for consumers. Diets high in products that contain higher levels of both the soy isoflavone, genistein, and its metabolite genistin, have been shown to affect breast tumor growth in a number of different models. In contrast, highly processed soy flour that does not contain isoflavones has no effect. Purified soy protein isolates are often processed to contain different concentrations of isoflavones, and their influence on mammary tumors is related to the amount of the isoflavone phytoestrogen, not the total amount of soy protein consumed [586].

Several epidemiological studies have shown that regular consumption of soy-based products, or other vegetables high in phytoestrogens, as part of a normal balanced diet can exert a protective influence with regards to later development of breast cancer. This effect has been studied extensively in China where soy intake is a regular part of the cultural diet. There, substantial evidence indicates that higher soy intake in adulthood or in adolescence is associated with a decreased risk of pre-menopausal breast cancer (OR = 0.41; 95% CI = 0.21–0.70 for soy intake; OR = 0.44; 95% CI = 0.26–0.73 for isoflavone intake [587]; OR = 0.68; 95% CI = 0.50–.93 for soy intake) [588]. Other studies have found protective effects of soy intake for both pre- and post-menopausal cancer, independent of receptor (ER and PR positive or negative) profile of the tumors (ORs ranging from 0.30 to 0.43) with a dose-dependent inverse relationship found across cancer subtypes (trend *p* < .0001) [589].

For Chinese women who were previously diagnosed with breast cancer, higher consumption of soy in its many forms found regularly in a woman’s diet, was correlated with decreased recurrence of cancer (OR = 0.68; 95% CI = 0.54–0.92) and longer survival (Or = 0.71; 95% CI = 0.54–0.87) [590]. Complicating the picture further is a study of Korean women who had previously been diagnosed and treated for breast cancer. Dietary soy intake was associated with a decreased rate of recurrence in women whose cancers were HER-2 negative (OR = 0.27; 95% CI = 0.13–0.57), and an increased rate of recurrence of cancer in women whose original tumors were HER-2 positive (trend *p* < .02) [591].

On the other hand, in a prospective study of women aged 43 to 55 years who had never been diagnosed with breast cancer but were considered to be at high risk, 6 months of dietary isoflavone (PTIG-2535, containing 150 mg genistein, 74 mg daidzein, and 11 mg glycitein) intake was associated with increased proliferation of breast cells. The effect was most pronounced in pre-menopausal women [592].

A series of reports from Japan looking at the relationship between soy intake and breast cancer risk have found an inverse relationship [593–595], with strong dose-response relationships being found for postmenopausal women (trend, *p* = .023 for soy intake and trend *p* = .046 for isoflavone intake) [596].

A study of Asian-American women living in California and Hawaii found significant decreases in breast cancer risk associated with greater soy intake during childhood (RR = 0.40 95% CI = 0.18–.83), adolescence (RR = 0.80 95% CI = 0.59–1.09), or adulthood (RR = 0.76 95% CI = 0.56–1.02) [597]. The protective effect of regular dietary soy intake during childhood was the strongest, and it was not mitigated when other variables like site of birth (Asian countries or U.S.), degree of continuing Asian lifestyle and cultural practices, reproductive factors or family history of breast cancer were factored into the analysis. In general, protective effects of dietary soy intake have been found to be strongest in association with childhood and early adolescent intake [598], especially in relationship to development of ER+/PR+ postmenopausal breast cancer (OR = 0.79; 95% CI = 0.65–0.96) [599]. One possible explanation for this association is that peri-pubertal exposures to genistein and other phytoestrogens may mimic the protective changes in breast development that are usually observed during the first pregnancy [600, 601].

Several studies have directly compared effects of consuming culturally appropriate soy diets in Asian and Western women. A 2012 meta-analysis that combined data from six studies found that regular dietary intake of soy during adolescence decreased the incidence of all later breast cancers (OR = 0.82 95% CI = 0.67–0.99), and was particularly effective in decreasing cancer incidence in pre-menopausal women (OR = 0.66 95% CI = 0.55–0.80). There was no reported difference in the effects of dietary intakes of soy during adolescence between Asian and American/European women [602]. On the other hand, a 2014 meta-analysis examining isoflavone intake in pre- and post-menopausal women from Asian and Western countries found protective effects of soy in both premenopausal (OR = 0.59; 95% CI = 0.48–0.69) and postmenopausal (OR = 0.59; 95% CI = 0.47–0.74) Asian women, with only very small and non-significant effects in both premenopausal and postmenopausal Western women [603]. A 2009 multiethnic study conducted in Hawaii demonstrated that the amount of soy in the diet might interact with associations between other phytoestrogens and protection against breast cancer. For Japanese Americans who had high soy content in their regular diets, there was a strong, significant but non-monotonic relationship for urinary genistein levels within the middle two quartiles (OR =0.88; 95% CI =0.78–0.99) with decreased risk of breast cancer. A similar strong relationship was not found for White women in the study who tended to eat diets lower in soy content [604]. Differences in responses between Asian and Western women may reflect both differences in the diet content as well as cultural differences in the ability to metabolize isoflavones, an effect that may result from both genetic differences as well as interactions with other dietary factors [584].

Data from studies on laboratory animals and cell culture models have indicated a more complicated story. In several studies, exposures to phytoestrogens have led to increases in mammary tumor proliferation and growth. In ACI rats, dietary exposure to total soy isoflavone content from conception through adulthood decreased incidence of mammary tumors in adult animals by 20% and multiplicity by 56%, while also decreasing the latency to tumor onset by 20% and almost tripling tumor volumes [605]. Exposure of Wistar rats to genistein from conception through weaning led to decreases in DMBA-induced tumor number, multiplicity and incidence at postnatal day 50 [606].

The soy phytoestrogens, genistein and daidzein, as well as their metabolites, cause oxidative DNA damage, a process that is thought to play a role in tumor initiation. Other data suggest that these two soy-based phytoestrogens may have opposing effects on the efficacy of the breast cancer drug, tamoxifen [607, 608].

The effects of the phytoestrogens may well be related to the particular components and doses in the diet [609], and cellular effects may vary depending on concentration and timing. In a study examining the effects of different types and concentrations of phytoestrogens on the expression of estrogen-dependent gene activity in human breast cancer cells grown in vitro (MCF-7 cells), low doses of genistein resulted in a pattern of expression that indicated increased cell proliferation, while somewhat higher concentrations led to increased apoptosis [610].

Isoflavones contained within natural soy flour have different effects than addition of isoflavones purified from the soy flour on gene expression patterns in induced tumors grown from MCF-7 cells in athymic nude mice. When mice were fed isolated isoflavones, the gene expression pattern was similar to that found in mice that had been treated with estradiol, while the pattern in animals treated with isoflavones included within a full soy flour were more like the negative control, suggesting an inhibitory effect of soy flour proteins on some of the proliferative effects of isolated isoflavones [611]. In addition to altering gene patterns associated with cell proliferation and carcinogenesis, genistein exposure also is a strong inhibitor of angiogenesis, an important process associated with tumor growth and metastasis. On the other hand, 30 days of treatment by gavage of peripubertal and adult ovariectomized rats with isoflavone supplements had no effect on cell proliferation or angiogenesis [612].

In cultured MCF-7 cells, the soy phytoestrogen daidzein slightly enhanced cell proliferation in the absence of natural estrogen (a possible model for post-menopausal breast cancer), while resveratrol (found in grapes and red wine) significantly decreased tumor cell proliferation [602]. These latter data are consistent with other studies finding anti-carcinogenic effects of resveratrol in several models [613, 614].

Concern has been raised about exposure of newborn babies to soy-based products, primarily through infant formulas. Although one study has shown that feeding only soy formula for the first 4 months of life was associated with a decrease in later development of breast cancer [615], animal studies have indicated deleterious effects of neonatal soy exposure on development of the female reproductive system and subsequent fertility [616].

##### Mycoestrogens (fungal estrogens)

Mycotoxins are compounds produced by several fungal species that contaminate agricultural and feed products, including corn silage and hay, both during before harvest and during later storage [617–619]. People are exposed to these compounds directly, by eating grains contaminated with the fungi, and indirectly, by eating meat from animals who have consumed contaminated feed [620].

Contamination of food by zearalenone (ZEA) and its natural metabolites has been associated with the development of precocious puberty, a known risk factor for breast cancer, in young girls [621, 622]. On the other hand, girls in the Jersey Girl Study who had higher urinary ZEA levels, resulting from recent intake of beef or popcorn, tended to be less likely to have reached the onset of breast development and to be of shorter stature. Almost 80% of girls in the study had detectable levels of mycoestrogens in their urine [623].

A case-control study of dogs revealed that higher dietary exposures to mycotoxins (aflatoxin G1 or G2) resulted in a significant increase in the number of mammary tumors (OR 2.74; 95% CI = 1.13–6.60 and OR 4.6; 95% CI = 2.2–7.8, respectively) [624].

In rat dams fed diets containing ZEA, both the compound and its metabolites crossed the placental barrier and also appeared in mother’s milk [625]. Exposure of female pups to environmentally relevant doses of ZEA during the last 2 (of 3) weeks of fetal development and the first few postnatal days resulted in long-term alterations in mammary gland development of the sort associated with increased risk for development of mammary tumors [626].

Rats treated on post-natal days 15–19 with ZEA and then with the carcinogenic substance, N-methyl-N-nitrosurea (MNU), at puberty developed fewer mammary tumors, with lower multiplicity, than matched controls treated only with the NMU carcinogen, although there was no difference in the latency to appearance of tumors in either group [627].

In cell culture models of human breast cancer, mycotoxins including Fusarin C and ZEA and their metabolites have been shown to be estrogenic [628]. For example, Fusarin C stimulates growth and proliferation of MCF-7 breast cancer cells via ER-mediated processes [629]. Similarly, ZEA also enhances proliferation of MCF-7 cells in vitro through estrogen-mediated pathways and activation of estradiol-induced gene expression [630, 631].

#### Natural, synthetic and genetically engineered hormones used in food production

##### Zeranol (Ralgro®)

The synthetic compound, zeranol (Ralgro®), is a potent non-steroidal growth promoter that mimics many of the effects of the natural hormone estradiol. Zeranol (ZER) is used extensively in the U.S. and Canada to promote rapid and more efficient growth rates in animals used as sources for meat [632].

As with the natural compound ZEA, ZER is a strongly estrogenic chemical as demonstrated by its ability to stimulate growth and proliferation of human breast tumor cells in vitro at potencies similar to the natural hormones, estradiol, and the known carcinogen, diethylstilbestrol (DES) [633]. A 2007 study demonstrated that adding ZER to cultured breast epithelial cells led to enhanced cell proliferation, accompanied by an upregulation or stimulation of the activity of protein disulfide isomerase, an enzyme whose activity is often increased in cancerous tissues [634].

Treatment of young adult female mice with ZER led to increased growth and branching of mammary glands, similar to what is found in mice treated with the natural hormone estradiol [635]. Increased ductile proliferation, in the absence of full maturation of the ducts through pregnancy and lactation, is associated with an increased risk for mammary (breast) tumors.

Brief (4-day) prepubertal exposure of mice or rats to either ZEA or ZER accelerated the onset of puberty, but did not affect development of the mammary gland structures through early adulthood [636, 637].

A series of studies examined estrogenic activity in normal breast epithelial cells and breast cancer cells treated with ZER. Abnormal cell growth was significant even at ZER levels almost 30 times lower than the FDA-established limit in beef [638]. Follow-up work demonstrated that ZER is comparable to natural estrogen (estradiol) and the synthetic estrogen diethylstilbestrol (DES) in its ability to transform MCF-10A human breast epithelial cells to a pre-cancerous profile in vitro [639]. Preliminary data indicate that serum from ZER-treated beef cattle can stimulate the proliferation of normal breast epithelial cells and the transformation of breast tumor cells in vitro [640, 641].

##### Bovine growth hormone (rBGH)/Recombinant Bovine Somatotropin (rBST)

Despite opposition from physicians, scientists and consumer advocacy groups, the Food and Drug Administration in 1993 approved Monsanto’s genetically engineered hormone product, recombinant bovine growth hormone (subsequently renamed recombinant bovine somatotrophin, rBST), for injection in dairy cows to increase milk production [642]. This hormone quickly found its way (without labeling) into the U.S. milk supply, and from there into ice cream, buttermilk, cheese, yogurt and other dairy products. Since its introduction, rBST has proven controversial because of its potential carcinogenic effects.

Drinking any type of cow’s milk noticeably raises body levels of insulin growth factor 1 (IGF-1), a naturally occurring hormone in both cows and humans. Injecting cows with rBST leads to an increase in IGF-1 levels in milk [643], although it is possible that the increased milk output by treated animals may dilute the excess production of hormone [644]. The content of IGF-1 in dairy milk is not altered by pasteurization [645].

Although the data are complex with some studies reaching different conclusions, several epidemiological studies have indicated a relationship between dairy consumption and breast cancer risk in pre-menopausal women [646]. Elevated levels of IGF-1, in particular, have been associated with increased risk of breast cancer [647–650]. A nested case-control study within a larger prospective study of American women found that pre-menopausal women with the highest levels of IGF-1 in their blood (drawn before cancer developed) were seven times as likely to develop breast cancer as women with the lowest levels when results were adjusted for plasma concentrations of the IGF binding protein [647]. No increased risk was noted in post-menopausal women. Three studies reported in 2005 by scientists in Sweden, the United Kingdom [651] and the United States [652] also showed an association between circulating levels of IGF-1 and the risk of breast cancer in pre-menopausal women.

One mechanism by which IGF-1 may raise risk in younger women is by increasing breast density in pre-menopausal women, a known risk factor for cancer [653]. In addition, laboratory studies have shown that IGF-1 can regulate the growth and increase the proliferation of breast cancer cells (MCF-7) grown in vitro [654] and decrease the death of mammary tumor cells in laboratory animals [655].

Proponents of rBST argue that IGF-1 is harmless because it occurs naturally in humans, is contained in human saliva and is broken down during digestion. However, animal evidence indicates that digestion does not break down IGF-1 in milk because casein, the principal protein in cow’s milk, protects IGF-1 from the action of digestive enzymes [656].

*Section summary:* When incorporated into regular nutritious diets, lignans and soy-based foods have been shown to be protective against breast cancer in numerous epidemiological studies. This protection is especially clear when dietary intake begins in childhood. On the other hand, both mycoestrogens and the stock animal growth enhancer zeranol are estrogenic in their interactions with human breast cells, including cells derived from cancers, in cell culture environments. The data are more ambiguous on possible effects of elevated IGF-1 levels, found after drinking cow’s milk.

### Non-EDC industrial chemicals

Not all chemicals associated with increased risk for breast cancer exert their effects through endocrine disrupting mechanisms. This section examines the literatures linking a increased risk for developing breast cancer to a few industrial chemicals, all of which have been determined to be carcinogenic by IARC (see Table 3). These compounds and/or the DNA adducts formed following exposures to the compounds, are directly mutagenic.

**Table 3:** Carcinogenicity classifications and sources of exposures of chemicals found in non-EDC industrial chemicals

Chemical	IARC	NTP	Source of exposures
Non-EDC Industrial Chemicals			
Benzene	1	K	Petrochemical solvent
Vinyl chloride	1	K	Monomer used in polyvinyl chloride (PVC) plastic
1,3-Butadiene	1	K	Byproduct of combustion
Ethylene oxide	1	K	Sterilizer, byproduct contaminant in some cosmetics

International Agency for Research on Cancer (*IARC*) classifications: 1 = Carcinogenic to humans, 2A = Probably carcinogenic to humans, 2B = Possibly carcinogenic to humans, 3 = Not classifiable as to its carcinogenicity to humans; U.S. National Toxicology Program (*NTP*) classifications: K = Known to be a human carcinogen, RA = Reasonably anticipated to be a human carcinogen. Source of exposure list contains most common exposure sources

**Table 4:** Carcinogenicity classifications of chemical exposures found in cigarette smoke

Chemical	IARC	NTP
Tobacco smoking: Active and passive		K
Polycyclic aromatic hydrocarbons (PAHs)		RA
Polonium-210		
Benzene	1	K
Vinyl chloride	1	K
1,3-butadiene	1	K
Nitrosamine ketone (NNK)		

International Agency for Research on Cancer (*IARC*) classifications: 1 = Carcinogenic to humans, 2A = Probably carcinogenic to humans, 2B = Possibly carcinogenic to humans, 3 = Not classifiable as to its carcinogenicity to humans; U.S. National Toxicology Program (*NTP*) classifications: K = Known to be a human carcinogen, RA = Reasonably anticipated to be a human carcinogen. Source of exposure list contains most common exposure sources

#### Benzene

Benzene is one of the largest volume petrochemical solvents currently in production, and global production rates are expected to continue to grow over the next several years. Chemical industries estimate that more than 46 million metric tons (more than 115 billion pounds) of benzene will be consumed globally by the year 2020 [657]. Exposures to benzene come from inhaling gasoline fumes, automobile exhaust, or cigarette smoke (primary and secondary) and from industrial burning. Benzene presents a serious occupational hazard for people exposed through their work in chemical, rubber, shoe manufacturing, oil and gasoline refining industries. Both the NTP and IARC have designated benzene as a human carcinogen [658, 659].

Epidemiological studies of the effects of benzene on breast cancer risk are difficult to conduct, mainly because exposures to benzene occur in conjunction with exposures to other chemicals that are also released in combustion and manufacturing processes. Also, few of the occupational studies focusing on chemical and automotive industries have included women in substantial numbers to draw meaningful conclusions. One study that did look at relevant occupations among female Chinese workers, examined incidence by occupation as standardized by general breast cancer incidence rates in Shanghai and the number of women in each occupation according to the 1982 census. The occupations in which elevated risks for breast cancer were found included scientific research workers (SIR + 3.3); medical workers practicing Western style medicine (SIR =14.7, 95% CI = 5.9–30.3) or Chinese-Western style medicine (SIR = 7.2; 95% CI = 4.4–11.4); as well as workers with expected lower exposures such as teachers, librarians and accountants (SIRs 2.3–2.7). In the same study, looking across professions, benzene exposure was associated with an elevated risk of breast cancer [660]. A study of a fairly small sample of women for whom researchers have benzene exposure data from their work at a shoe factory in Florence, Italy, also supports a relationship between exposure to benzene and later development of breast cancer [661].

The largest study implicating benzene and associated chemicals comes from an occupational study looking at men who have been diagnosed with breast cancer. Men who had worked in professions that involved exposures to gasoline fumes and combustion had significantly increased rates of breast cancer. The effect was most pronounced among men who started at their jobs before the age of 40 [662].

Benzene administration to laboratory mice induces mammary tumors. Mice exposed to benzene have frequent mutations of genes that are responsible for suppressing the development of tumors [663, 664].

#### Vinyl chloride

Manufacturers use polyvinyl chloride (PVC) extensively to produce food packaging, medical products, appliances, cars, toys, credit cards and rainwear. When PVC is made, vinyl chloride may be released into the air or wastewater. Vinyl chloride has also been found in the air near hazardous waste sites and landfills and in tobacco smoke.

Vinyl chloride was one of the first chemicals designated as a human carcinogen by the NTP [665, 666]. Vinyl chloride has been linked to increased mortality from breast and liver cancer among workers involved in its manufacture [667, 668]. In the large prospective cohort California Teachers Study, exposure to vinyl chloride was associated with increased breast cancer risk. Analyses of subsets within the cohort reveal significant associations between vinyl chloride exposure (for highest quintile vinyl chloride exposure) and ER+/PR+ tumors (HR = 1.08; 95% CI = 0.98–1.19), and in women who had never, or were not currently, using HRT (HR = 1.27; 95% CI =1.04–1.54) [669]. In a case-control study of male breast cancer patients, all of whom had lived at Camp LeJeune during the decades when the drinking water was contaminated with several toxic solvents, exposures to vinyl chloride was associated with higher risk of developing breast cancer (OR = 1.20 (95% CI = .16–5.89) and earlier onset of the disease (OR = 2.14; 95% CI =0.31–14.81) [670].

Animals exposed long-term to low levels of airborne vinyl chloride show an increased risk of mammary tumors [671].

#### 1,3-butadiene

1,3-butadiene is an air pollutant created by internal combustion engines and petroleum refineries. It is also a chemical used in the manufacture and processing of synthetic rubber products and some fungicides. In addition, 1,3-butadiene is found in tobacco smoke.

The EPA determined that 1,3-butadiene is carcinogenic to humans, with the main route of exposure being through inhalation. Women working in the synthetic rubber industry who had high exposures to 1,3-butadiene had increased risk of dying from breast cancer (RR = 2.6 m 95% CI = .9–7.3) [672]. The NTP classifies 1,3-butadiene as a known human carcinogen [673].

Data from research on animals indicate that females may be more vulnerable to the carcinogenic effects of 1,3-butadiene [674], which is known to cause mammary and ovary tumors in female mice and rats. This pollutant produces even greater toxic effects in younger rodent populations [675].

#### Ethylene oxide

Ethylene oxide is a fumigant used to sterilize surgical instruments and is also used in some cosmetic products [676]. Ethylene oxide is classified as a human carcinogen [677, 678] and one of 221 chemicals identified by researchers at the Silent Spring Institute as being associated with mammary tumors in animals [197].

Scientists from the National Institute for Occupational Safety and Health (NIOSH) studied breast cancer incidence in 7576 women exposed to ethylene oxide while working in commercial sterilization facilities. They found an increased incidence of breast cancer among these women in direct proportion to their cumulative exposure to ethylene oxide [679]. Although there are contradictory data in the recent literature [678], other occupational studies support the finding that exposure to ethylene oxide is associated with increased risk for breast cancer in women [680, 681].

Studies in which human breast cells grown in vitro were exposed to low doses of ethylene oxide demonstrated that the chemical exposure resulted in a significant increase in damage to the cells’ DNA [682]. These findings are supported by results of a study examining gene mutations in mammary tumors induced in mice by exposures to ethylene oxide. Common mutations included those in the tumor suppressor gene, p53, and the cell proliferation regulatory gene, H-ras [664].

*Section summary:* Epidemiological studies of both men and women exposed occupationally to benzene or vinyl chloride have higher risk for developing breast cancer. Limited human also indicate that exposures to 1,3-butadiene also have an increased risk for breast cancer, while the evidence supporting this relationship is more robust for ethylene oxide.

### Tobacco smoking: active and passive

Increasing evidence indicat4es that exposure to the many chemicals included in tobacco smoke, both through active (first hand) and passive (second hand) means, can increase risk for developing breast cancer. We discuss this literature in this section. While exposures to smoke are often clustered with alcohol consumption and other lifestyle factors, in this category we only focus on tobacco smoke exposures as these are from chemicals polluting the environment, and the exposures are often involuntary.

Tobacco smoke contains polycyclic aromatic hydrocarbons (PAHs), as well as hundreds of other chemicals [683], including three known human carcinogens (polonium-210, a radioactive element; benzene; and vinyl chloride) as well as 1,3-butadiene and nicotine-derived nitrosamine ketone (NNK), all of which are known to cause mammary tumors in animals. NNK is a tobacco-specific carcinogen that has been shown to increase tumor cell proliferation and the transformation of healthy breast epithelial cells into cancer cells [684–686], at least in part via the nicotinic acetylcholine receptor [687] (see Table 4).

A large study of California teachers revealed an increased risk of breast cancer among smokers, particularly those who began smoking during adolescence (HR = 1.17; 95% CI = 1.05–1.30), at least 5 years before their first full-term pregnancy (HR = 1.13; 95% CI = 1.00–1.28), or who were longtime or heavy smokers (HR = 1.32; 95% CI = 1.10–1.57) [688]. Several earlier studies also suggest that women who begin smoking cigarettes as adolescents face increased risks of breast cancer [689–693].

Results from the Canadian National Breast Screening Study indicated that increased incidence of breast cancer was associated with longer duration of smoking (RR = 1.50; 95% CI = 1.19–1.89), number of cigarettes smoked per day (for 40 cigarettes/day: RR = 1.20; 95% CI = 1.00–1.44), and cumulative exposure to cigarette smoke (40 pack-years: RR = 1.17; 95% CI = 1.02–1.34) [694]. Similar results were recorded in reports from two large prospective studies: the Nurses Health Study [695] and the WHI study [696], which involved approximately 110,000 and 80,000 participants, respectively.

Although several more recent studies have reported that beginning smoking before a first full-term pregnancy (independent of age of onset of smoking) may make a woman increasingly susceptible to later diagnosis with breast cancer [691, 695, 697], a 2011 meta-analysis of 23 relevant research papers did not find a statistically significant relationship [698]. Complicating this picture is a report from the EPIC cohort reporting that the most important impact of active cigarette smoking was the number of pack-years (1 pack-year = 20 cigarettes/day for a full year) smoked from menarche to first full-term pregnancy (HR = 1.73, 95% CI = 1.29–2.32 for every increase of 20 pack-years). On the other hand, the number of pack-years smoked following menopause was significantly associated with a decreased risk for developing breast cancer (HR = 0.53; 95% CI = 0.34–0.82) [699]. Very different results were reported in a large study from the African American Breast Cancer Epidemiology and Risk (AMBER) study: as compared with women who never smoked, higher pack-years of active smoking in pre-menopausal women was associated with a decreased risk of breast cancer (OR = 0.80; 95% CI = 0.68–0.96), while higher pack years smoked in active postmenopausal smokers was associated with an increased risk of breast cancer (OR = 1.16; 95% CI = 1.01–1.33) [700]. The postmenopausal effect was strongest in women developing ER+ cancers. In post-menopausal women, an inverse relationship was found between active smoking and mammographic breast density, with the effect being magnified for women who started smoking before the age of 16 (OR = 0.79; 95% CI = 0.64–0.96) [701]. Lower breast density is associated with lower risk of developing breast cancer [702].

A population-based case control study examined effects of active smoking and risk of developing breast cancer based on whether tumors were luminal (ER+ and/or PR+) or basal (ER-, PR-, HER2-) types. Ever smoking led to an increased risk of developing luminal-type cancer (OR = 1.12; 95% CI = 0.92–1.33), but not basal-type, an effect that was most pronounced in Black women. Another study looking at inflammatory breast cancer incidence reported effects of active smoking on luminal (OR = 2.37; 95% CI = 1.24–4.52), but not other types of breast cancer [703]. However increased smoking duration was associated with an increased risk of developing basal type (OR = 1.51; 95% CI = 1.19–1.93), but not luminal, cancers [704].

Ethnic differences were reported in a study of Mexican and U.S. non-Hispanic white women. For Mexican women, a significant increase in breast cancer risk was found for former smokers (OR = 1.43; 95% CI = 1.04–1.96 vs. never smokers), and this effect was increased for former smokers with a history of alcohol consumption (OR = 2.30; 95% CI = 1.01–5.21). For U.S. non-Hispanic white women, current smoking of more than 20 cigarettes a day was associated with increased risk (OR = 1.61; 95% CI = 1.07–2.41). There were no significant effects found for U.S. Hispanic white women [705].

A prospective cohort study of 186,150 female AARP members, 7486 of whom developed breast cancer, found an increased risk of developing breast cancer in current active smokers (OR = 1.19; 95% CI = 1.10–1.28) as well as former active smokers (OR = 1.07; 95% CI = 1.01–1.13). For current smokers, the effect was significant in women with no family history of breast cancer (OR = 1.24; 95% CI = 1.15–1.35), but not in women with a family history. Later age of menarche was also associated with higher risk for developing breast cancer in active smokers (age of menarche x smoking status interaction, *p* < .03) [706].

Several recent studies have examined the effects of smoking at the time of breast cancer diagnosis and subsequent outcomes. Bérubé reported that smoking at the time of diagnosis led to an increase in all-cause mortality, as well as a significant increase in mortality from breast cancer (HR = 1.33; 95% CI = 1.12–1.58) [707]. Similar effects on breast-cancer mortality were reported (HR = 1.10; 95% CI = 0.73–1.68) in women diagnosed with localized breast cancer [708]. Continued active smoking after diagnosis was associated increase in breast cancer-related deaths (HR = 1.72; 95% CI = 1.13–2.60) [709].

In 309 female ER+ breast cancer patients being treated with aromatase inhibitors, smoking before surgical treatment for their disease was associated with increased numbers of breast cancer events (e.g., recurrence, new breast cancer diagnosis, metastasis; HR = 2.97; 95% CI = 1.44–6.13), distant metastases (HR = 4.19; 95% CI = 1.81–9.72), and mortality (HR = 3.52; 95% CI = 1.59–7.61). There was no relationship between smoking and breast cancer-related outcomes in women undergoing other forms of adjuvant therapy [710].

Two recent studies have examined smoking status and breast cancer outcomes in men. In a pooled case-study consortium with 2378 cases of male breast cancer in Florida, Cook et al. found no evidence of association between smoking status, pack-years, duration, or age at initiation of smoking and risk of developing breast cancer [711]. Another study of male breast cancer cases in Florida examined survival rates following diagnosis and stratified their analysis by race-ethnicity and socioeconomic status. Overall, as compared with never smokers, current smokers had higher mortality rates (HR = 1.63; 95% CI = 1.23–2.16), although there was no effect for past smokers who had given up the habit. There was a dose-response relationship between amount smoked and mortality risk (trend, *p* < .001). Similar effects were found for both White (but not Black) and non-Hispanic (but not Hispanic) men [712].

In addition to effects of active smoking on breast cancer incidence and mortality, a growing literature implicates exposures to second hand smoke (passive smoking) to increased risk for the disease. Until recently, more evidence linked secondhand smoke than active smoking to breast cancer risk. Current evidence suggests that both exposures increase breast cancer risk by about the same amount, even though women who are exposed to secondhand smoke receive a much lower dose of carcinogens than do active smokers [699, 713, 714]. Researchers at Japan’s National Cancer Center reported the results of a study involving 21,000 women ages 40 to 59. They found that the risk of breast cancer was elevated in pre-menopausal women who were either active smokers (RR = 3.9; 95% CI = 1.5–9.9) or exposed to second-hand environmental smoke (RR = 2.6; 95% CI = 1.3–5.2) [715]. Other major studies, including the WHI, support the finding of a link between extensive exposure to passive smoking lasting more than 10 years and increased risk for breast cancer (HR = 1.32; 95% CI = 1.04–1.67) [696]. Exposures to passive smoke at home, but not at work, increases risk of developing breast cancer (OR = 1.30; 95% CI = 1.05–1.61) and the amount of exposure at home is linked in a dose-response fashion (*p* = .009) [716].

A meta-analysis of eight studies of Chinese women exposed to passive smoking who were never active smokers themselves showed a significant increase in risk of developing breast cancer (OR = 1.67; 95% CI = 1.27–2.21) [717]. A more detailed analysis of Chinese women who had never smoked showed a significant effect of more than 4 pack-years of exposure to passive smoke (OR = 1.71; 95% CI = 1.17–2.50). The effect was found in women with ER+/PR+ tumors, but not for other tumor subtypes [718].

In trying to understand the mechanisms by which active and/or passive smoking might affect risk for developing breast cancer, several gene expression studies have been conducted. Studies exploring links between smoking and breast cancer incidence, recurrence and mortality have identified several polymorphisms associated with increased risk. The most consistent data implicate a ‘slow acetylator’ n-acetyltransferase 2 (*NAT2*) phenotype [719, 720], although specific polymorphisms of the *BRCA1* [719, 721] and the *CYP1A1* and *COMT* [719] genes have also been reported to be associated with increased incidence and/or mortality in active smokers.

Other physiological disruptions resulting from exposures to smoke include damaging the structure and function of the ovaries, thereby lowering estrogen levels in pre-menopausal women. While lower levels of estrogen would decrease breast cancer risk, at the same time carcinogens in cigarette smoke would increase risk of developing breast cancer [722].

A cross sectional study which is a part of a large, on-going prospective project (the EPIC cohort), examined the association between tobacco smoking and sex hormone levels in post-menopausal women, whose ovaries are no longer the major source of their of circulating hormones. Smoking was related to higher levels of testosterone, estradiol and other steroid hormones [723]. The increased levels of circulating estradiol were only statistically significant for women who were considerably overweight. By itself, obesity is a known risk factor for postmenopausal breast cancer. Adipose tissue is the main site of aromatization of testosterone to estradiol in men and postmenopausal women, and increased adipose tissue can thus contribute to increased circulating estrogens. Greater activation of breast fat cell metabolic pathways by tobacco-containing chemicals may enhance the development of breast cancer [724].

*Section summary:* There is now a substantial literature indicating that past and current active cigarette smoking is associated with a higher risk for developing breast cancer. For women who are smokers at the time of diagnosis, there is also an increased risk in mortality from breast cancer. These effects are complicated by interactions with race/ethnicity, history of alcohol consumption and subtype of breast cancer being evaluated.

### Shift work, light-at-night and melatonin

In 2007, IARC concluded that shift work is ‘probably carcinogenic to humans’ based in large part on the growing association between shift working and increased incidence of breast cancer [725] (see Table 5). Several occupational studies have demonstrated that women who consistently work night shifts have increased breast cancer risk, although not all reports have found evidence for this relationship. Methodological differences between studies, including varied definitions of “shift work” and “night,” as well as lack of consistent attention to confounding factors may explain some of the differences in results between individual studies [726, 727].

**Table 5:** Carcinogenicity classifiations and exposure sources of light-at night and radiation

Exposure	IARC	NTP	Use
Shift Work, Light-at-Night	PR		Shift work or ambient light pollution
Ionizing Radiation	K	K	Diagnostic medical tests; nuclear medicine procedures; nuclear power plants, research protocols
Non-ionizing radiation (electromagnetic fields)			Lighting, computers, cell phones and other electronic sources

International Agency for Research on Cancer (*IARC*) classifications: 1 = Carcinogenic to humans, 2A = Probably carcinogenic to humans, 2B = Possibly carcinogenic to humans, 3 = Not classifiable as to its carcinogenicity to humans; U.S. National Toxicology Program (*NTP*) classifications: K = Known to be a human carcinogen, RA = Reasonably anticipated to be a human carcinogen. Source of exposure list contains most common exposure sources

Associations between long-term (> 20–30 years) night-shift work and breast cancer were reported in a 2008 comprehensive review of 13 studies [728]. Four other reviews that included meta-analyses reached similar conclusions although the strength of the associations varied considerably. Based on analysis of 13 studies, Megdal and colleagues reported an aggregated estimate of breast cancer risk (RR) for both airline attendants and others working on night shift work as 1.48 (95% CI = 1.36–1.61) [729]. Kamdar and colleagues included 15 case-control and cohort studies examining the possible relationship between night shift work and breast cancer risk, and reported a pooled RR of 1.21 (95% CI = 1.00–1.47) for individuals with any experience with night shift work as compared with those without such experience [730]. He et al. included a more heterogeneous group of 28 studies that examined circadian rhythm disruptions, but defined in various ways (shift work, short sleep duration, occupation as flight attendant, light-at-night exposure). They reported an aggregate RR of 1.14 (95% CI = 1.08–1.21), with similar RRs when analyses were limited to just studies examining shift work (RR = 1.19; 95% CI = 1.08–1.32) or light-at-night exposure (RR = 1.12; 95% CI = 1.12–1.12), but a considerably higher risk when studies of just flight attendants were analyzed (RR =1.56 (95% CI = 1.10–2.56). Shorter duration of sleep did not confer a change in risk for developing breast cancer in this analysis [731]. Lin, et al. analyzed 16 prospective cohort studies and reported an aggregate RR of 1.09 (95% CI = 1.02–1.17) for night shift workers compared with day workers. A linear trend (*p* = 0.010) was found for increased exposure length (< 5, 5–10, 10–20 and >20 years) and risk for developing breast cancer [732]. On the otherhand, a new meta-analysis of 10 prospective studies, including a total of 1.4 million women, found no effects on breast cancer risk for engaging in any shift work (RR = 0.99; 95% CI = 0.95–1.03), for 20 or more years of night shift work (RR = 1.01; 95% CI = 0.93–1.10), or for 30 or more years of night shift work (RR = 1.00; 95% CI = 0.87–1.14) [733]. A more recent evaluation of this meta-analysis called into question several methodological criteria and data interpretations within the report [734].

A record linkage study of occupation and cancer in Britain calculated that for 2012, the population attributable factor (PAF) of night shift work may account for 4.5% (95% CI = 3.2–5.9) of breast cancer diagnoses (1957 cases; 95% CI = 1395–2547) and deaths (552 cases; 95% CI = 393–724) [735]. A similar analysis in 2015 calculated a PAF for shift work of 5.7% (95% CI = 0.0—11.9) of U.S. women being diagnosed with breast cancer (attributable breast cancer cases = 11,777; 95% CI = 0–24,625) [736].

In a study of Danish nurses, effects of night shift work on breast cancer risk were greatest for women who worked rotating hours that include the overnight, as opposed to evening shift (OR = 1.8; 95% CI = 1.2–2.8) and for those who worked 12-h shifts that alternated day and night work, as compared to shorter work periods (OR = 2.9; 95% CI = 1.1–8.0) [737].

Risk of developing breast cancer increased for women who worked night shifts for more than 4.5 to 5 years (OR = 1.40; 95% CI = 1.01–1.92), and for those who regularly engaged in night work for at least 4 years prior to their first pregnancy (OR = 1.95; 95% CI = 1.13–3.35), therefore before the time when their mammary cells had fully differentiated [738].

The most thoroughly studied mechanism to explain these effects of night shift work is the light-at-night (LAN) hypothesis [739]. Increasing exposure to light, especially bright indoor light, at times outside of normal daylight hours, decreases secretion of melatonin by the pineal gland. Normal high levels of melatonin at nighttime are important for regulation of both pituitary and ovarian hormones, for suppressing the local production of estrogen resulting from aromatization of androgens in breast tumor cells, and for maintaining normal metabolic profiles and body weight [740–743].

Clinical studies have demonstrated that there is a decrease in the peak amount of melatonin secreted in women with metastatic cancer, as compared with healthy women, and larger tumors are associated with lower levels of melatonin [740]. Blind women who are completely unable to perceive the presence of environmental light, and concommitantly have no daily decreases in melatonin levels, have significantly lower risk of diagnosis of breast cancer than do blind women who do perceive light and have regular changes in melatonin secretion over the normal 24-h cycle (OR = 0.52; 95% CI = 0.27–1.01) [744].

One proposed pathway by which reduced melatonin might affect breast cancer risk is enhancement of the production or secretion of estradiol and other ovarian hormones. Nagata et al. reported that postmenopausal women who worked night shifts that went beyond midnight had significantly increased serum concentrations of estradiol both during their night shift phases and when they rotated to regular day awake periods, as compared with controls who did not engage in late night shift work [745]. Davis et al. tested urinary levels of 6-sulfatoxymelatonin (the major metabolite of melatonin), LH, FSH and estrone conjugate across sleep and work cycles for premenopausal nurses working both day and night shifts. As compared with nurses working day shifts, in night shift workers, LH and FSH levels were both significantly higher (35 and 38% higher, respectively) while 6-sulfatoxymelatonin levels were significantly decreased (69% lower); no significant differences were found in estrone conjugate [746]. However, one study that examined the possible link between changes in melatonin levels and changes in reproductive hormone levels did not find a relationship once other factors like age, menstrual status and body mass index were factored into the analysis [747].

In rodent models, higher levels of melatonin are associated with decreased incidence and size of mammary tumors, and when they do occur, the latency period of tumor development is lengthened [739]. In human mammary tumors that had been grafted into mice, perfusion with blood taken from women at night (when melatonin is high) decreased proliferation and growth of mammary tumors, as compared to the use of samples collected during the day when melatonin levels are naturally lower [748].

Mechanistically, night pulses of melatonin enhance the activity of endocrine, metabolic and immune-related pathways that can prevent the development of cancer [749]. These protective effects of melatonin are mediated by epigenetic changes in many of the genes involved in regulation of cell growth and proliferation, as well as in the synthesis and activation of the estrogen receptor [750]. Genes that are associated with the regulation of the daily melatonin cycle also regulate other pathways that may be involved in the development of breast cancer. Structural variation in one such gene, *Per3*, is associated with higher breast cancer rates in young women [751]. *Per2*, another gene associated with the control of daily rhythms, is also poorly regulated in many women with breast cancer, with normal structure and expression of this gene being associated with lower effectiveness of estradiol in altering cellular activity. In healthy cells, *Per2* also may act directly as a tumor-suppressor gene, decreasing the activity of pathways associated with tumor formation [752]. A rare polymorphism of the *CLOCK* gene has been associated with an increased risk for developing breast cancer (OR = 3.53; 95% CI +1.09–11.42), and there was a positive interaction between the presence of this genotype and night shift work on risk for developing breast cancer (*p* = .02) [753]. However, a case-control study that evaluated 100 SNPs of 14 clock-related genes in interaction with shift work history found no associations for any of the SNPs [754].

Other recent studies have greatly complicated the light-at-night and breast cancer story. Relationships between night shift work and melatonin levels may be mediated by race/ethnic background. In a large population-based study of Chinese women, no association between shift work and breast cancer incidence was reported [755]. Asian and Asian-American women who work night shifts have less melatonin suppression than their white counterparts [756]. Effects of night shift work may also be limited to specific breast tumor types. In a population based case-control study of night shift workers, adjusting for chronotype (mid-sleep point on days when participants could choose when to sleep), resulted in increased risk of invasive (vs. in situ) tumors (OR = 1.23; 95% CI = 1.05–1.99) and increased risk of premenopausal ER+/PR+ tumors (OR = 1.44; 95% CI = 1.05–1.99) [757].

Several authors have proposed that factors associated with night shift work, beyond decreases in melatonin levels, need to be considered in understanding better the links with increased risk for breast cancer. Other possible consequences of shift work, including phase shift sleep disruption, lifestyle factors, changes in metabolism, desynchronization between central neural and peripheral systems, or decreased vitamin D production, may also be linked to increased cancer rates. These factors need to be studied as both single and possibly interacting factors in altered risk for developing breast cancer [758–762].

Finally, as with several endocrine disrupting chemicals, light-at-night decreases significantly the effectiveness of major chemotherapeutic agents used in the treatment of breast cancer. In rat models with MCF-7 breast cancer cells xenografts, addition of dim light exposures during the dark phase of the cycle led to decreased melatonin secretion during the dark phase, decreased latency to tumor progression, increased tumor growth, and complete resistance to both tamoxifen and doxorubicin [763, 764].

*Section summary:* Extensive experience with night shift work, and therefore higher exposure to light-at-night (LAN), has been shown to increase risk for breast cancer, although there may be ethnic differences in this response. The most studied underlying mechanism for the effect of LAN exposures is the accompanying change in patterns of melatonin secretion. Other lifestyle and physiological factors associated with shift work have also been proposed to alter risk for developing breast cancer.

### Radiation

Exposure to ionizing radiation, from both military and medical sources, is the best known and longest established environmental cause of breast cancer in both women and men. Exposures early in life, during childhood through adolescence, are particularly important. Data for potential links between electromagnetic fields, or non-ionizing radiation, and breast cancer are mixed and inconclusive.

#### Ionizing radiation

Ionizing radiation is any form of radiation with enough energy to break off electrons from atoms (to ionize the atoms). This radiation can break the chemical bonds in molecules, including DNA molecules, thereby disturbing their normal functioning. X-rays and gamma rays are the only major forms of radiation with sufficient energy to penetrate and damage body tissue below the surface of the skin.

Among the many sources of ionizing radiation are traditional X-rays, computed tomography (CT) scans, fluoroscopy and other medical radiological procedures. Sources of gamma rays include emissions from nuclear power plants, scientific research involving radionuclides, military weapons testing and nuclear medicine procedures such as bone, thyroid and lung scans [765].

In 2005, the National Toxicology Program classified X-radiation and gamma radiation as known human carcinogens [766] (see Table 5). Although some scientists challenge this premise [767], most agree that no safe dose of radiation has been identified [768, 769]. Radiation damage to genes is cumulative over a lifetime [770]. Repeated low-dose exposures over time may have the same harmful effects as a single high-dose exposure.

Exposure to ionizing radiation is the best- and longest-established environmental cause of human breast cancer in both women and men. The link between radiation exposure and breast cancer has been demonstrated in atomic bomb survivors [771–774]. Rates of breast cancer were highest among women who were younger than age 20 when the United States dropped atomic bombs on Hiroshima and Nagasaki [773]. In addition, Ron et al. reported a significant association between ionizing radiation exposure and the incidence of male breast cancer in Japanese atomic bomb survivors [775].

Ionizing radiation can increase the risk for breast cancer through a number of different mechanisms, including direct mutagenesis, genomic instability [776, 777] and changes in breast cell micro-environments that can lead to damaged regulation of cell-cell interactions within the breast [778–780]. Ionizing radiation not only affects cells that are directly exposed, but it can also alter the DNA, cell growth and cell-cell interactions of neighboring cells, referred to as the ‘bystander effect.’ [767, 781]. A G2 micronucleus assay of blood samples from asymptomatic women carrying the *BRCA1* mutation have deficits in many of these cell processes and a heightened sensitivity to the effects of radiation exposures, as compared with samples from healthy women without the mutation [782].

*Interactions Between Radiation and Other Factors.* There are a number of factors that may interact with radiation to increase the potency of its carcinogenic effects. Some of these factors include a woman’s age at exposure, genetic profile and possibly estrogen levels. Studies of women exposed to military, accidental or medical sources of radiation have demonstrated clearly that children and adolescents who are exposed are more seriously affected in their later risk for breast cancer than are older women [769]. In addition, recent genetic data indicate that women with some gene mutations (e.g., *ATM, TP53* and *BRCA1/2*) are more likely to develop breast cancer and may be especially susceptible to the cancer-inducing effects of exposures to ionizing radiation [71, 783–785].

Studies using animal and in vitro human breast tumor cell culture models have demonstrated that the effects of radiation on mammary carcinogenesis may be additive with effects of estrogens [786–788]. This is of particular concern given the widespread exposure to estrogen-mimicking chemicals in our environment and the multiple sources of ionizing radiation.

##### Occupational exposures

Female radiology technologists who had sustained daily exposures to ionizing radiation demonstrated an increased risk of breast cancer for those women who began working during their teens or, independent of age, working in the field before the 1940s, when exposure levels were substantially higher than they have been in more recent decades [789, 790]. Follow-up of this cohort for another decade revealed an increased mortality rate for technologists who began work before 1950, with a significant trend (*P* = .01) for correlation with earlier year beginning work. Technologists who began working before 1950 and had worked for at least 5 years had an increased mortality rate from breast cancer (HR = 2.25; 95% CI = .95–6.68) [791]. In a subset of this cohort who had worked with fluoroscopically guided interventional procedures, there was an increased incidence of breast cancer compared to technologists who had not engaged in this work (HR = 1.166; 95% CI = 1.02–1.32), but no effect on mortality from breast cancer [792]. The susceptibility of radiologists to later diagnosis of breast cancer may be affected by common variants in genes that are involved in the metabolism of circulating estrogens [793].

A review and analysis of all existing related studies found that women who work as airline flight attendants had increased levels of breast cancer [794]. A meta-analysis of seven studies examining cancer incidence in flight attendants reported an elevated incidence of breast cancer (SIR = 1.40; 95% CI = 1.19–1.65) [795]. Factors that could explain this increase may include lifestyle and reproductive histories, light-at-night exposures, as well as increased exposures to cosmic (atmospheric) ionizing radiation.

##### Medical radiation: risks and benefits

*Medical X-rays:* Use of X-rays to examine the spine, heart, lungs, ribs, shoulders and esophagus also exposes parts of the breast to radiation. X-rays and fluoroscopy of infants irradiate the whole body [796]. Decades of research have confirmed the link between radiation and breast cancer in women who were irradiated for many different medical conditions, including tuberculosis [105], benign breast disease [106, 107], acute postpartum mastitis [108], enlarged thymus [109, 110], skin hemangiomas [111], scoliosis [112], Hodgkin’s disease [113–116], non-Hodgkin’s lymphoma [117], acne [118], and prophylactic dental care [119].

Anytime use of diagnostic chest X-rays before the age of 50 years in women carrying the *BRCA1/2* gene mutations is associated with an increased risk of breast cancer (*BRCA1* OR = 1.16; 95% CI = .64–2.11; *BRCA2* OR = 1.22; 95% CI = .62–2.42) [797].

Evidence from almost all conditions suggests that exposure to ionizing radiation during childhood and adolescence is particularly dangerous with respect to increased risk for breast cancer later in life [75, 120, 121, 126] and that there is a significant dose-response relationship between the dosage of childhood radiation and the increased incidence of breast cancer (trend *p* < .001) [798]. Importantly, use of radiation in pediatric medicine leads to higher effective dose for children than for adults given the equivalent radiation exposure, a reflection of their smaller body sizes [799, 800].

*Computed Tomography (CT) Scans:* There is credible evidence that medical X-rays (including mammography, fluoroscopy and CT scans) are an important and controllable cause of breast cancer [119, 801]. Although there has been a substantial decrease in exposures to ionizing radiation from individual X-rays over the past several decades, there has been a six-fold increase in exposure to medical sources of radiation from the mid-1980s through 2007, with an annual increase of 16%, primarily from the increased use of CT scans and nuclear medicine [802, 803]. In 2007, approximately 72 million CT scans were conducted in the United States [804]. When a CT scan is directed to the chest, the individual receives the equivalent radiation of 30 to 442 chest X-rays [805]. Modeling estimates have indicated that use of chest CTs and CT angiography in 2007 alone will lead to an additional 5300 cases of lung and breast cancer within the next two to three decades [804]. Other modeling suggests that 1 in 150 women who are 20 years old when they undergo CT angiograms of the chest, and 1 in 270 women of all ages having the procedure, will subsequently develop cancers of the chest, including breast cancer [806].

CT angiography, a source of comparatively high radiation to the chest, has been associated with a significant increase in risk for developing breast cancer, especially in pre-menopausal women [807, 808].

*Mammography:* Many experts believe that the low-dose exposures to radiation received as a result of mammographic procedures are not sufficient to increase risk for breast cancer. However, damage from lower-energy sources of X-rays, including those used in mammography, cannot be predicted by estimating risk from models based on higher doses [75, 809]. Evidence indicates that the lower-energy X-rays provided by mammography resulted in substantially greater damage to DNA than would be predicted by those models. Evidence also suggests that risk of breast cancer caused by exposure to mammography radiation may be greatly underestimated [808].

As with other risk factors for breast cancer, both age at exposure and the individual’s genetic profile influence the degree of increased risk for disease in women exposed to multiple mammograms. For example, women who had multiple mammograms more than 5 years prior to diagnosis had an increased risk for breast cancer, but the effect was only statistically significant for women whose first mammograms began before age 35 [119].

This age effect is of particular concern, as it is often recommended that high-risk women, including carriers of either of the *BRCA* mutations, begin annual mammography screening at age 25 to 30. But, young women with these mutations are actually more vulnerable to the cancer-inducing effects of early and repeated mammograms. This increased vulnerability has been reported in women with *BRCA1/2* mutations [71, 72] as well in women with other relatively uncommon variations in genes known to be involved in the process of DNA repair [75]. For women with *BRCA2* mutations, meta-analysis of seven articles found low-dose exposures from either mammography or chest X-rays led to an increase risk for breast cancer (OR = 1.3; 95% CI = .9–1.8). Exposure before the age of 20 increased risk (OR = 2.0; 95% CI = 1.3–3.1), as did greater than 5 years of exposures (OR = 1.8; 95% CI = 1.1–3.0) [67]. Diagnostic radiation has been shown to increase risk for developing breast cancer in a dose-dependent manner [73].

The detrimental risks from mammography might also be heightened in older women, whose breast epithelial cells have gone through several decades of cell division. Cells derived from older women’s breast tissue were more sensitive to the DNA-damaging effects of low-energy radiation, increasing the likelihood of later conversion to cancerous cells [810].

In 2009, the U.S. Preventive Services Task Force (USPSTF) recommended against the use of routine mammography screening before the age of 50 (Nelson, [811]; USPSTF, 2009) but supported the use of biennial screening between the ages of 50 and 75 [811]. These recommendations were based on models using a number of factors, including positive and negative test results and the psychological consequences of those results on women; number of follow-up imaging procedures and biopsies; actual diagnoses; and, ultimately, mortality rates from breast cancer. Not considered in the analysis was the contribution of radiation from either single or repeated mammograms or other follow-up tests [812]. Several analyses suggest that for women over the age of 40 who are not at high risk, the trade-offs between diagnostic efficacy of mammography and radiation exposure lean more in the favor of regular mammography screening [813–815]. In 2016, the USPSTF updated their recommendations for women between 40 and 49 years, leaving the decision on whether or not to start mammographic screening up to the individual woman [816]. As women are now facing the need to make their own decisions about whether to undergo routine screening mammography, it is critical that both physicians and women are better educated about mammography’s potential harms, along with its potential benefits [72, 817].

*Radiation therapy:* Some studies suggest that doctors and patients should carefully evaluate the risks and benefits of radiation therapy for survivors of early-stage breast cancer, particularly older women. Women older than 55 derive less benefit from radiation therapy in terms of reduced rate of local recurrence [818] and may face increased risks of radiation-induced cardiovascular complications [819], as well as secondary cancers such as leukemias and cancers of the lung, esophagus, stomach and breast [820, 821]. Using NCI’s Surveillance, Epidemiology and End Results (SEER) data, researchers showed a 16-fold increased relative risk of angiosarcoma of the breast and chest wall following irradiation of a primary breast cancer [822]. Angiosarcomas of the breast are associated with relatively poor prognosis [823].

More recent data indicate that women younger than 45 who received the higher radiation exposure associated with post-lumpectomy radiotherapy (as compared to post-mastectomy radiation) had a 1.5–2.5-fold increase in later contralateral breast cancer diagnoses. This effect was especially prominent in younger women with a substantial family history of breast cancer [824–826]. Bernstein et al. studied a cohort of women, nested within the large WECARE study, who had developed contralateral breast cancer (as compared to breast cancer patients who did not develop contralateral breast cancer). They found main effects for both gene status and treatment, with significant elevations for *BRCA1/2* carriers (RR = 4.5; 95% CI = 3.0–6.6), and ever treatment with radiotherapy (RR = 1.2; 95% CI = 1.0–6.6), but no significant interaction between the two factors [74].

##### Non-ionizing radiation (electromagnetic fields)

Electromagnetic waves are a type of low frequency, non-ionizing radiation without enough energy to break off electrons from their orbits around atoms and ionize the atoms. Microwaves, radio waves, radar and radiation produced by electrical transmission are examples of radiation sources that generate electromagnetic fields (EMF). Fluorescent lighting, computers and many other types of wired and wireless electronic equipment (e.g., cell phones) all create electromagnetic fields of varying strengths.

Both IARC and the National Institute of Environmental Health Sciences (NIEHS) EMF Working Group have classified EMF exposures as a possible human carcinogen based on the scientific literature related to EMF and childhood leukemias [827]. More recently, data have suggested a link between EMF exposure, especially from cell phone use, and development of brain cancer and acoustic neuromas, although the strength of these connections remain controversial [828]. Concensus has been even more been more difficult to reach about the relationship between EMF and breast cancer.

Although many epidemiological or occupational studies have not found significant relationships between exposures to EMF and risk for breast cancer, others have reported data supporting these effects [829, 830]. Methodological issues may account for some of the discrepancies, given the relatively small effects that are found and the ubiquitous nature of “background” EMF in our daily lives [831].

Kliukiene et al. reported an increased risk of breast cancer among Norwegian female radio and telegraph operators exposed to radiofrequency (one type of EMF) and extremely low frequency EMF. Pre-menopausal women showed an increased risk of estrogen-receptor-positive tumors (OR = 1.78; 95% CI = 0.59–5.41) and post-menopausal women had an increased risk of estrogen-receptor-negative tumors (OR = 2.37; 95% CI = 0.88–6.36) [832].

In an occupational study that looked at women in job setting with the potential for high, medium or low electromagnetic exposures, high exposures were associated with an increased risk for developing breast cancer (OR = 1.43; 95% CI = 0.99–2.09). Pre-menopausal women appear to be at higher risk (OR = 1.98; 95% CI = 1.04–3.78) than post-menopausal women (OR = 1.33; 95% CI = 0.82–2.17) [833].

Studies of residential and occupational EMF exposure found a significant increase (OR = 1.58; 95% CI = 1.30–1.92) in breast cancer risk among women of all ages living near high-voltage power lines. Similar effects were found for women with ER+ and ER– tumors. Occupational exposure also increased risk (OR = 1.13; 95% CI = 0.91–1.40) [834]. Women younger than age 50 who were exposed to EMF both at home and at work had a modest increase in risk of breast cancer [835].

Nevertheless, meta-analyses of 15 studies concluded that there is no clear relationship between EMF exposure and breast cancer in women (OR = 0.99; 95% CI = 0.90–1.09) [836]. Another meta-analysis that examined a subset of published studies that specified mode of exposure reported a small increase in breast cancer rates in premenopausal women associated with increased residential exposure to EMF (OR = 1.18; 95% CI = 1.02–1.37) [837].

Although breast cancer is rare in men, numerous occupational exposure studies point to a connection between EMF exposure and male breast cancer [838–842].

In the laboratory, EMF can cause increased mammary tumors in animals and proliferation in systems in which human breast cell tumors are grown in culture. Importantly, effects in rodents are found in some strains of animals but not others, indicating that subtle differences in genetic background might make some animals more susceptible to the carcinogenic effects of EMF [843]. In an in vitro cell system, EMF exposure of human breast tumor (MCF-7) cells led to an activation of genes that have been associated with the induction of metastasis in breast cancer cells [844].

*Section summary:* Exposure to ionizing radiation is a known cause of increased risk for breast cancer. Victims of military use of nuclear bombs have increased risk, as do women who had X-ray treatments for medical purposes, especially when they were young. Women carrying the *BRCA1/2* mutations are particularly susceptible to the effects of X-rays, including those emitted by routine mammography.

More mixed results come from studies of women exposed to non-ionizing radiation, either because of occupational or residential exposures.

## Discussion and conclusions

In the 8 years since we last published an extensive review of the relevant literature, hundreds of new papers have appeared addressing the link between exposures to environmental toxicants and an increased risk for developing breast cancer; the majority of the studies support the existence of this link for the agents discussed in this review. Not only has the corpus of the literature expanded in size over the past several years, but it has also been enhanced by greater depth, breadth, and complexity. The growing literature on developmental exposures to EDCs and later development of breast cancer is especially strong.

Epidemiological data strongly support the link between increased risk of developing breast cancer and early developmental exposures to DES, DDT and radiation, as well as adult exposures to oral contraceptives and HRT. A growing literature also implicates engaging in night shiftwork as an important factor leading to increased risk for breast cancer. On the other hand, a substantial literature examining the effects of consuming soy products and lignans as part of a regular diet, especially starting early in life, indicates they can have a protective effect against later development of breast cancer.

Animal and other in vitro models support the hypothesis that many other chemicals found in commonly used consumer products, as well as in our air, water and dust, all are associated with increased risk for predisposing mammary tissue to develop tumors. These data support the strong links described above for EDCs. Data from epidemiological studies suggest connections between exposures and later development of cancer, although methodological limitations often constrain the conclusions that can be drawn. Of particular concern for most epidemiological studies in this field is the lack of direct measurement of toxicant exposure levels in individuals, especially in the years (or decades) prior to diagnosis of the breast cancer [845]. As the literature has documented clearly, there is often a long latency between exposures and diagnosis, and earlier developmental exposures can be especially powerful in affecting development of breast cancer, even decades later [457]. To enlarge the body of relevant work, it will be important for large cohort studies to regularly collect exposure information across much of the lifespan, and to develop the technologies necessary to quantify exposure levels, biomarkers, and health outcomes at large-scale levels [845, 846].

Importantly, through animal, cell culture, high throughput and other non-epidemiological models, mechanisms are being elucidated by which exposures to various toxicants may lead to increased risk for developing cancer. This literature has been slow to develop, because regulatory toxicological studies examining reproductive and developmental consequences of exposures to various drugs or potential toxicants have not required examination of mammary tissue endpoints [847]. There are not standardized protocols for determining appropriate times of exposures, ranges of doses, or mammary gland endpoints to study and later potential carcinogenic, genotoxic, or endocrine disrupting effects of these exposures. In order to claim more definitively the connections between the many chemicals that have been implicated in increased risk for development of breast cancer and causal links to the disease, it will be important to develop a series of endpoints to be studied routinely. Critical endpoints to be evaluated include altered mammary gland development; activity of various biomarkers including PR, HER, other endocrine factors; and different subtypes of various hormone receptors, each of which can have different effects on cellular activity when activated [848].

Despite these critical methodological limitations and concerns, the breadth and strength of the evidence cited in this review, when taken as a whole, reinforce the conclusion that exposures to a wide variety of toxicants – many of which are found in common, everyday products and byproducts – can lead to increased risk for development of breast cancer. As concluded by the reports of the Presidential Cancer Panel [4] and the Interagency Breast Cancer and Environment Research Coordinating Committee [2], it is critical to recognize the growing literature demonstrating connections between exposures to environmental toxicants and later development of disease, including breast cancer, and to prioritize prevention both at the research and the public health levels.

## Abbreviations

2,4,5-TP: 2,4,5-trichlorophenoxypropionic acid
4-NP: 4-nonylphenol
ABP: 4-aminophenyl
AhR: Aryl hydrocarbon receptor
AR: Androgen receptor
BPA: Bisphenol A
CHDS: Child Health and Development Study
DCIS: Ductal carcinoma in situ
DDE: Dichlorodiphenyldichloroethylene
DDT: Dichlorodiphenyltrichloroethane
DEHP: Di(2-ethylhexyl)phthalate
DEP: Diethyl phthalate
DES: Diethylstilbestrol
DiBP: Di-i-butyl phthalate
EDC: Endocrine disrupting compound
EMT: Epithelial-mesenchymal transition
EPA: Environmental Protection Agency
ER: Estrogen receptor
ERR: Estrogen-related receptor
FDA: Food and Drug Administration
HE: Heptachlor epoxide
HHA: Heterocyclic aromatic amines
HRT: Hormone Replacement Therapy
IARC: International Agency for Research on Cancer
IGF-1: Insuling growth factor-1
IVF: In vitro fertilization
LAN: Light-at-night
MEHP: Mono(2-ethylhexyl)phthalate
MMP: Monomethyl phthalate
NAT2: N-acetyltransferase 2
NCI: National Cancer Institute
NHANES: National Health and Nutrition Examination Survey
NMR: Non-monotonic response
NNK: Nitrosamine ketone
NOAEL: No-observed-adverse-effect-level
NTP: National Toxicology Program
OMC: Octyl-methoxycinnamate
PAH: Polyaromatic hydrocarbon
PBDE: Polybrominated Diphenyl Ether
PCB: Polychlorinated biphenyl
PFC: Polyfluorinated chemical
PFOA: Perfluorooctanic acid
PFOS: Perfluorooctanic sulfate
PhIP: 2-amino-phenylimidazo-[4.5.-b]pyridine
PR: Progesterone receptor
PVC: Polyvinyl chloride
rBST: Recombinant Bovine Somatotropin
SEER: Surveillance, Epidemiology and End Results
SNP: Single-nucleotide polymorphism
T4: Thyroid hormone
TCDD: 2,3,7,8-tetrachlorodibenzo-para-dioxin
TEB: Terminal end buds
TOFT: Tissue Organization Field Theory
WHI: Women’s Health Initiative
YES: Yeast estrogen screen
ZEA: Zearalenone
ZER: Zeranol
